# Host Plant Association and Distribution of the Onion Thrips, *Thrips tabaci* Cryptic Species Complex

**DOI:** 10.3390/insects13030298

**Published:** 2022-03-17

**Authors:** Roberto Carlos Loredo Varela, József Fail

**Affiliations:** Department of Entomology, Institute of Plant Protection, Hungarian University of Agriculture and Life Sciences, 44 Menesi ut, 1118 Budapest, Hungary; rloredovarela@hotmail.com

**Keywords:** pest thrips, breeding sites, finding sites, distribution, leek-associated lineages, tobacco-associated lineage

## Abstract

**Simple Summary:**

Onion thrips, *Thrips tabaci* Lindeman, 1889, is a key insect pest of several cultivated plant species around the world. Current genetic evidence suggests the existence of three lineages within the species; these lineages are different from each other in several aspects, including reproductive mode, ecological parameters, orthotospovirus transmission efficiency, host plants and distribution range. Despite its importance as a crop pest and the fact that it is one of the most studied thrips species, there is not a comprehensive review of plants in which evidence of breeding occurs among the lineages and the whole species complex. Since identifying the breeding sites of onion thrips has a direct impact on successful pest management strategies, in this paper, we aim to provide a literature review about the host plant association and distribution of the three onion thrips lineages. The results indicate that leek-associated 2 is the most widespread lineage by number of host plants and distribution; leek-associated 1 lineage is primarily found on onion crops and with localised distribution and the tobacco-associated lineage is only reported from tobacco in few locations. In addition, we present a list of host plants for the species, regardless of lineage: 391 plant species from 64 families.

**Abstract:**

Onion thrips, *Thrips tabaci* Lindeman, 1889 (Thysanoptera: Thripidae) is a pest of economic importance traditionally treated as a polyphagous, cosmopolitan single species. Recent genetic evidence, however, suggests that it is rather a cryptic species complex of three lineages referred to by their host association and displaying different biological and ecological characteristics: leek-associated 1, leek-associated 2 and tobacco-associated. This study reviews host plant associations and distribution of the lineages of this cryptic species complex and discusses its consequences from an agronomical perspective. Overall, leek-associated 2 lineage has the broadest host range, including major crops from different plant families, and it is the only lineage with a confirmed worldwide distribution. Leek-associated 1 lineage shares some host plants with leek-associated 2. It is often found in *Allium* crops and its geographic distribution is limited to a few dozen countries. Finally, tobacco-associated lineage has only been collected from tobacco and their associated weeds in central and east Europe, and the Middle East. Additionally, this work presents a list of 391 plant species on which breeding and development of *T. tabaci* occurs, regardless of lineage. These host plant species belong to 64 different families, most importantly Asteraceae, Fabaceae, Brassicaceae, Poaceae, and Solanaceae.

## 1. Introduction

The onion thrips, *Thrips tabaci* Lindeman, 1889 (Thysanoptera: Thripidae) represents a significant threat to agricultural production worldwide because of five main reasons: first, this species injures over 30 major crops [1]. Second, onion thrips has a global distribution, as its presence is reported in over 120 countries and territories [2]. Moreover, this species is adapted to a wide range of environmental conditions [3,4], although rarely abundant in wet tropical zones [5]. Third, *Thrips tabaci* is highly invasive due to its small size, cryptic behaviour, polyphagy, short generation time, presence of sexual and asexual populations, high reproductive capacity, and ability to disperse to neighbouring fields [6] and greenhouses [7,8] and to be transported along the international trade of agricultural products [9]. Fourth, it can damage crops either by direct feeding of the first and second instar larvae and adults on different plant organs [4] or by transmitting plant orthotospoviruses, namely, *Tomato spotted wilt virus* (TSWV) [10], *Iris yellow spot virus* (IYSV) [11], *Tomato yellow ring virus* (TYRV) [12] and *Alstroemeria yellow spot virus* (AYSV) [13]. Fifth, pesticide resistance has been observed in several onion thrips populations around the world, leading to additional damage [14,15].

The end results of direct and indirect damage of this species are the negative impact on food security and revenue losses worldwide. For instance, onion thrips direct feeding on onion crops reduces both yield and quality of product in several regions worldwide [16]. In addition, there are reports in which onion thrips act as a primary vector of orthotospoviruses. For example, TSWV outbreaks cause considerable losses in tobacco crops across central and eastern Europe [17,18]; in another case, IYSV causes annual losses of tens of millions of US dollars to onion crops in western USA alone [19].

Other characteristics of winged adult thrips are their highly dispersive behaviour; even though adult thrips are weak flyers, they can easily be airborne and readily carried by the wind or other transportation means [20]. In the case of adult onion thrips, they have been collected from hundreds of different plant species at a national level [21,22,23,24]. However, this does not mean that every plant from which adult thrips are recorded is suitable for their development [25,26]. As a matter of fact, an accurate number of host plants for many thysanopteran species has not yet been established for a number of reasons. Most importantly, published records of onion thrips and other thysanopteran species frequently fail to distinguish between the terms *finding site* and *host* *plant* [5,25]. In *finding sites* only adult thrips are found and their presence might be explained by different events such as an accidental landing event and/or the plant serves only as an occasional feeding site [5,25]. On the other hand, a *host plant* serves as breeding site for the species; hence both larvae and adults occur [5,25]. Additional factors influencing the incorrect host plant association of thrips species are: first, the incorrect identification of thrips species and second, morphological identification of immature stages is often overlooked, prioritising adult-stage identification [25].

Furthermore, most of the available literature considers onion thrips as a single species with a broad host plant range [21,22]. Nonetheless, recent publications reveal the existence of a cryptic species complex within *T. tabaci* formed by three lineages with different biological, ecological and genetic traits [27]. Based on a study of onion thrips populations in Poland by Zawirska [28], proposed two biotypes within this species based on host preference, distribution within the country, TSWV vector efficiency and a single morphological difference in the larval phase. The *communis* biotype has thelytokous reproductive mode in which 100% of unfertilised eggs develop into females, broad distribution and numerous host plants, larvae have a distinct crest on the posterior edge of tergite IX and are very inefficient vector of TSWV. In contrast, the *tabaci* biotype has arrhenotokous reproductive mode in which 100% of unfertilised eggs develop into males and fertilised eggs give rise to females, localised distribution and a host range that is narrower than that of the *communis* type. Moreover, the *tabaci* biotype is a very efficient vector of TSWV and the second instar larva has no crest on the posterior edge of abdominal tergite IX. Additionally, the author described two strains within the *tabaci* biotype: one preferring tobacco plants and the other showing a preference for *Lamium* spp. [28].

In the past decades, DNA-based identification techniques have been incorporated in the study of thrips. These methods overcome some difficulties presented when using morphological identification methods as they can be used regardless of developmental stage [29], polymorphism and sex of the individuals [30]. Comparisons of small segments of genomic regions, especially mitochondrial Cytochrome c Oxidase subunit I (mtCOI), have been used for: (1) identification of species and cryptic (sub)species [29,31]; (2) to establish phylogenetic relationships within cryptic (sub)species complex [27]. In addition, the use of molecular methods has advanced our knowledge of some biological and ecological phenomena, for example: the gene flow among cryptic thrips species [32,33], occurrence of sympatric populations within cryptic species complex [34] and studying the cost of sexual reproduction [35]. Additionally, molecular methods have been integrated into studies of agronomical importance; for instance identification of cryptic and/or sympatric pest thrips populations and their correlation with parameters such as orthotospovirus vector competency [36,37], performance on cultivated host plants [38] and resistance to insecticides [14,15,39].

Phylogenetic analysis of partial mtCOI gene sequences of onion thrips, collected from leek and tobacco fields, suggest that this species can be categorised into three main evolutionary lineages based on host association: leek-associated 1 (L1), leek-associated 2 (L2) and tobacco-associated (T) [27]. Additionally, several studies found correlation between this cryptic species complex and their reproductive mode, concluding that lineages L2 and L1 are associated with thelytokous and arrhenotokous reproductive modes, respectively [40,41]. The tobacco-associated lineage (T) has arrhenotokous reproductive mode [42]. However, occurrence of deuterotoky (reproductive mode in which unfertilised eggs produce both male and female individuals) has been reported once in the USA [43] and another time in Hungary [44].

Significant differences regarding TSWV vector transmission efficiency among lineages of the onion thrips cryptic species complex have been reported [10,36,45]. The thelytokous leek-associated lineage has high variation in vector efficiency, mostly reported with no or marginal capacity to transmit the orthotospovirus [10,36,45,46,47]; however, other authors reported high capacity to transmit TSWV [36]. The arrhenotokous leek-associated lineage is identified as a low to moderate TSWV transmitter [10,45,48]. In contrast, the arrhenotokous tobacco-associated lineage is a highly effective TSWV transmitter [10,28]. Nonetheless, other factors might be involved in the transmission efficiency by this vector, including specific virus–vector interactions [37]. Transmission efficiency among *T. tabaci* lineages and other orthotospovirus species vectored by this species largely remain unknown [12,13,49].

The importance of finding breeding sites of this cryptic species complex is relevant since it has a direct impact on pest management measures carried out in agroecosystems. Finding the breeding sites for this species helps to identify which plant species are involved in its survival at specific times and locations [50,51,52]. Examples of integrated pest-management strategies that require precise information regarding breeding sites of this insect include: (1) decision making regarding the use of insecticides [18]; (2) weed elimination approaches before, during and after growing season [51,52]; (3) selection of intercrops [53]; (4) selection of *trap* crops [54]; (5) selection of *banker* plants to support the establishment of biological control agents [55]; (6) identification of suitable plants for the establishment of *push–pull* strategies [56]; (7) furthermore, knowing the breeding sites of onion thrips has critical implications for the control of the viral diseases vectored by this insect. Orthotospoviruses can only be acquired by onion thrips when larvae feed on infected plant material [10,50,51,52].

This review paper has two aims. The first objective is to establish host association and distribution among the three known lineages of the onion thrips cryptic species complex. The second objective is to list the host plants of onion thrips, regardless of the lineage involved, and to check whether these host plant species are also hosts of the currently known four orthotospovirus species transmitted by onion thrips.

## 2. Materials and Methods

### 2.1. Host Plant Range and Distribution in the Onion Thrips Cryptic Species Complex

A literature search of published papers before September 2021 and GenBank accessions (https://www.ncbi.nlm.nih.gov/genbank/ (accessed on 13 March 2021)) was carried out to determine the current state of knowledge of host association and distribution among the onion thrips cryptic species complex. As most of the published information for identification of lineages within the onion thrips cryptic species complex are based on direct sequencing and then phylogenetic analysis or PCR-based techniques using a portion of the mtCOI gene, only information using this gene region was included.

In addition, a literature search was carried out to find the host association of the onion thrips cryptic species complex based on their reproductive mode. Most of the information available relates to the thelytokous reproductive mode of the L2 lineage [40,42], while L1 and T lineages have arrhenotokous reproductive mode [38,40,42]. As a consequence, this assessment includes information in which onion thrips asexual reproductive mode was established by observing the sex ratio in the progeny of virgin females under controlled laboratory conditions. This review also includes information about thelytokous reproductive mode obtained from laboratory rearing studies started from individuals in which mating status were not previously checked (mated/unmated) but no males were detected after several generations [36,40,57]. As thelytoky has only been reported from the L2 lineage of onion thrips, we may consider onion thrips individuals reproducing with thelytoky belonging to the L2 lineage.

Worldwide distribution of the lineages of this cryptic species complex is presented on figures that were created using the online available tool Mapschart (https://mapchart.net/index.html, accessed on 13 March 2021) with information provided in the Invasive species compendium [2] (https://www.cabi.org/isc/datasheet/53746, accessed on 13 March 2021) and references cited in the first two tables.

### 2.2. Host Plant Range of Onion Thrips Regardless of Lineages

The definition of host plant used in this review is the one given by Mound [25]: “*a plant on which the insect is able to rear its young*”. As a result, host association of onion thrips was confirmed by reviewing published literature in which immature stages were found to thrive on a particular plant species and then identified to species level. The published methodologies to associate onion thrips and determined host plant can often be divided into three categories: (1) direct identification of the larval stage previously collected in a particular plant species using morphological identification keys [58]; (2) alive larvae were collected from a particular plant species then reared to adulthood; afterwards, adults were morphologically identified to species level [52,59], and (3) field collection of adults; subsequently, these individuals were reared on diverse plant species under controlled environmental conditions and identified using either morphological or molecular methods [38,60]. Thrips larvae may unintentionally be transported from plant to plant by the wind, or they can accidentally fall from other plant species, especially on vegetation growing beneath trees [61]. To reduce these potential error factors, we included, when available, multiple references to support a given plant as a host plant. We also selected, when available, those articles presenting results from multiple sampling events describing the collection site and/or studies where the authors presented information from rearing onion thrips on different plant material under controlled conditions. However, we also included references in which no detailed information was presented regarding the collection site.

Once host plants of onion thrips were established, we compared existing host plant reports of TSWV [62,63], IYSV [11,52,63,64,65], TYRV [12,63,66] and AYSV [13] to check whether these plant species are also hosts of the currently known orthotospovirus species *T. tabaci* vectors.

The plant species names used in this document are the up-to-date accepted names given by the World Flora Online consortium (http://www.worldfloraonline.org/ (accessed on 13 March 2021)) and Plants of the world online (https://powo.science.kew.org/ (accessed on 13 March 2021)). Additionally, subspecies and plants identified only to the genus level (e.g., *Alstroemeria* sp.) are considered separate botanical entities and they are also listed in this review.

## 3. Results and Discussion

### 3.1. Host Plants and Distribution of the Onion Thrips Cryptic Species Complex Determined by Molecular Identification Tools

**Figure 1:**
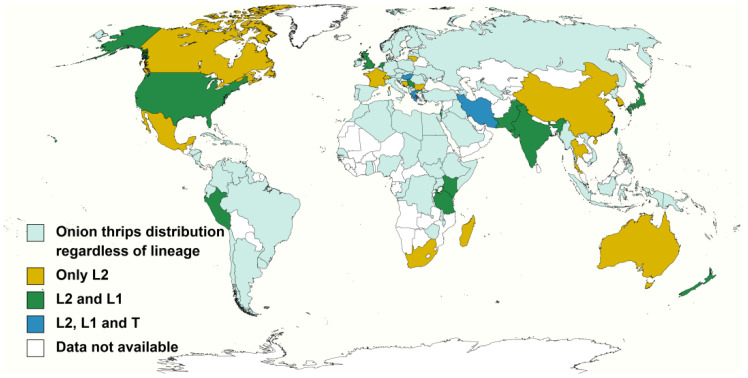
Evidence of the distribution of the onion thrips cryptic species complex determined by molecular identification methods. Onion thrips lineages based on host association proposed by Brunner et al. [27].

Molecular identification methods based on sequence variation of a region of the mtCOI gene have been applied to onion thrips specimens collected in 51 plant species from 29 countries (Table 1 and Figure 1).

**Table 1:** Finding sites and host plants of onion thrips cryptic species complex determined by molecular identification methods.

Plant Species	*Thrips tabaci* Lineages	
	L2	L1
**Amaryllidaceae**	-	-
*Allium ampeloprasum* L.	A [27,34]; N/A [40]	A [27,34]; N/A [40,67]; R [42,68]
*A. cepa* L.	A [30,36,41,69,70,71]; L [33]; N/A [40,72,73]; R [38]	A [30,41,70]; A, L [33]; N/A [40,42,67,72,74,75]; R [76]
*A. fistulosum* L.	A [77,78]; N/A [15,40]	A [77,78]; N/A [15]
*A. sativum* L.	N/A [40]	-
*A. tuberosum* Rottler ex Spr.	A [69,77]	A [77]
*A. × wakegi* Araki	N/A [40]	-
*Allium* spp.	N/A [79]	-
**Apiaceae**	-	-
*Ammi majus* L.	A, L [80]	-
*Coriandrum sativum* L.	N/A [42]	-
*Eryngium* sp.	N/A [73]	-
**Asparagaceae**	-	-
*Asparagus officinalis* L.	N/A [15,40,73]	-
**Asteraceae**	-	-
*Arctotheca calendula* (L.) Levyns	A [36]	-
*Bidens pilosa* L.	-	N/A [81]
*Chrysanthemum* sp.	A [36]	-
*Cichorium endivia* L.	N/A [73]	-
*Dittrichia viscosa* (L.) Gr.	A [29]	-
*Erigeron annuus* (L.) Pers.	N/A [42]	-
*Liatris* sp.	N/A [40]	N/A [40]
*Santolina chamaecyparissus* L.	N/A [42]	-
**Balsaminaceae**	-	-
*Impatiens* sp.	A [36]	-
**Boraginaceae**	-	-
*Echium plantagineum* L.	A, L [80]	-
**Brassicaceae**	-	-
*Brassica carinata* A.Braun	-	N/A [72]
*B. oleracea* L.	A [71]; N/A [79]; R [38,42]	R [32,33,38]
*Brassica* spp.	N/A [79]	N/A [81]
*Raphanus raphanistrum* subsp. *sativus* (L.) Domin	N/A [40,79]	-
*R. raphanistrum* L.	N/A [79]	-
**Caprifoliaceae**	-	-
*Lonicera caprifolium* L.	N/A [42]	-
**Caryophyllaceae**	-	-
*Dianthus* sp.	N/A [40]	N/A [40]
**Cucurbitaceae**	-	-
*Cucumis sativus* L.	A [29]; N/A [67]	-
**Ebenaceae**	-	-
*Diospyros kaki* L.f.	A, L [14]; N/A [40]	-
**Fabaceae**	-	-
*Medicago sativa* L.	A [36]; A, L [80]	-
*Phaseolus vulgaris* L.	N/A [67]; R [36,70]	-
*Pisum sativum* L.	N/A [73]	N/A [81]
*Trifolium repens* L.	N/A [40]	-
*Vicia faba* L.	R [14,15,39,40,77]	R [40,77]
**Gentianaceae**	-	-
*Eustoma grandiflorum* (Raf.) Sh.	N/A [40]	-
**Lamiaceae**	-	-
*Ocimum basilicum* L.	N/A [74]	N/A [74]
**Malvaceae**	-	-
*Gossypium hirsutum* L.	A, L [80]	-
**Poaceae**	-	-
*Secale cereale* L.	N/A [79]	-
**Rosaceae**	-	-
*Dasiphora fruticosa* (L.) Rydb.	N/A [42]	-
*Filipendula vulgaris* Moench	N/A [42]	-
*Fragaria×ananassa* (Duchesne ex Weston) Duchesne ex Rozier	N/A [73]	-
*Sorbaria sorbifolia* (L.) A.Br.	N/A [42]	-
**Rutaceae**	-	-
*Citrus unshiu* (Yu.Tanaka ex Swingle) Marcow	N/A [40]	-
**Solanaceae**	-	-
*Capsicum annuum* L.	N/A [67]	-
*Nicotiana tabacum* L.	A [27,30]	A [27]
*Solanum lycopersicum* L.	-	N/A [81]
*S. nigrum* L.	A [36]	-
*S. tuberosum* L.	A [36]; N/A [40]	-
**Urticaceae**	-	-
*Urtica dioica* L.	A [29]	-
**Verbenaceae**	-	-
*Verbena tenuisecta* Briq.	A [80]	-
**Plant species not determined**	A [29,82]; N/A [31,40,81]; N/A [83] (GenBank KM535733, KM529437 and KM528653); N/A [84] (KF534480-KF534483)	A [29]; N/A [31,81]; N/A [85] (GenBank accessions FN546159-FN546164)

Notes: L2 = Leek-associated 2 and L1 = Leek-associated 1 lineages according to Brunner et al. [27]. Developmental stage in which onion thrips individuals were collected from the plant species under field conditions and then used to form laboratory colonies and/or used directly for genetic analysis. A = Adult; L = Larvae; N/A = Not available. R = onion thrips individuals were reared for one or more generations under controlled environmental conditions on various plant species. - = There are no data.

L2 lineage was the most common lineage encountered since: (1) it usually represented the most abundant lineage within the collected samples [69,71,80]; (2) it was collected from 48 plant species, including major crops such as: leek, onion, garlic, Welsh onion, oriental garlic, asparagus, endive, cabbage, carnation, cucumber, persimmon, alfalfa, pea, cotton, strawberry, pepper and potato; as well as different weed species. Additionally, L2 laboratory populations are routinely reared on several plant materials including onion, cabbage, common bean and faba bean. Besides, (3) L2 lineage was recorded in all 29 nations surveyed, including areas in which only this lineage is reported such as Australia [36,80] and mainland China [69]. Other countries reporting L2 populations were: Bosnia–Herzegovina [29], Bulgaria [27], Canada [83], France [40], Greece [27], Hungary [42], India [70], Iran [30], Israel [40], Japan [40], Kenya [81], Lithuania [84], Madagascar [72], Mexico [73], Netherlands [40], New Zealand [73], Pakistan [31], Peru [73], Serbia [67], South Korea [40], South Africa [82], Switzerland [27], Taiwan [73], Tanzania [72], Thailand [73], United Kingdom [29] and United States [71].

L1 lineage was the second most common lineage as it was found in 15 plant species sampled in 16 countries and regions including Greece [27], Hungary [42], India [70], Iran [30], Israel [40], Japan [77], Kenya [81], Netherlands [40], New Zealand [68], Pakistan [31], Peru [74], Serbia [67], Taiwan [73], Tanzania [72], UK [85] and USA [33]. As the L2 lineage, L1 lineage individuals were found in high numbers in onion-like crops mainly in leek, bulb onion, Welsh onion and Oriental garlic. There are also reports of L1 specimens collected from *Brassica* spp., other cultivated species and weeds. Moreover, germinated faba beans, onion and leek are regularly used as food source for L1 laboratory colonies.

Lastly, there are far fewer reports about the host association and distribution of the T lineage; therefore it is not included in Table 1. T lineage individuals were only collected and identified to lineage level from tobacco plants [27,30,42,75]. The T lineage distribution is exclusively reported in Central and East Europe and Middle East countries; interestingly, this localised distribution overlaps with the areas in which onion thrips are the main thysanopteran pest species responsible of causing important economic damage to tobacco crops [3,18,27,28,42]. Outside these regions, onion thrips are not considered a key pest of tobacco [86].

Despite the predominance of the T individuals on tobacco plants, a few onion thrips adults identified within the L2 and L1 lineages have been collected from tobacco plants in Switzerland and Greece [27] and Iran [30]. Host preference tests confirm that arrhenotokous tobacco-associated group is the only onion thrips population able to grow and develop normally on tobacco leaves. Thelytokous leek-associated and arrhenotokous leek-associated populations were not able to grow and develop when reared on tobacco leaves [10]. On the contrary, arrhenotokous tobacco-associated individuals were able to grow and develop normally in leek and onion [10]. There is no information available regarding whether L2 and L1 lineages are able to thrive on tobacco inflorescences.

The information presented in Table 1 and Table 2 shows that different lineages might share several hosts or finding places. Moreover, some studies report differences in the performance of these lineages when feeding on various food sources. The most dramatic example is the high adaptation of the T lineage to tobacco crops and the null or very low survivorship reported for L1 and L2 lineages on this solanaceous crop [10,27,30]. For other crops, these differences in host adaptation are not so pronounced and require detailed comparison of life table and demographic parameters, as well as competition experiments among lineages under controlled conditions. For instance, Li et al. [38] compared net reproductive rate, mean generation time, intrinsic rate of natural increase, finite rate of increase and population doubling time between thelytokous L2 and arrhenotokous L1 populations developing in both cabbage and onion. Their results pointed out that L1 lineage outcompeted L2 lineage when growing and developing in onion plants. The opposite occurred in cabbage as L2 outperformed L1 lineage. These sorts of performance comparisons between lineages in other host plants will eventually allow us to have more complete information about host preference/avoidance and consequently, will impact decision making at the farm level to control this pest.

Additionally, the occurrence of sympatric populations of onion thrips lineages has been reported in nature, particularly in *Allium* species. For instance, thelytokous L2 and arrhenotokous L1 populations have been found coexisting in onion [41], leek [27,34], oriental garlic [77] and Welsh onion crops [77,78]. In addition, Table 1 and Table 2 show that adult and immature individuals of different lineages and reproductive modes have been collected and/or successfully reared on a particular plant species. This suggests that sympatric populations of onion thrips lineages can form more often than previously believed. There are several implications associated with the aforementioned coexistence; one consequence might be an ongoing gene flow among lineages, previously thought to be reproductively isolated, that might affect their biological and ecological characteristics. For instance, Li et al. [32] reported successful mating between arrhenotokous L1 males and thelytokous L2 females; they also confirmed gene transfer from sexual L1 lineage to asexual L2 offspring during laboratory interbreeding tests. Nevertheless, the effective fertilisation of L2 eggs with sperm coming from L1 males was calculated at a low rate (1.9%) [32]. Similarly, Jacobson et al. [33] reported evidence supporting gene transfer among the L1 and L2 lineages, albeit on rare occasions, occurring in onion fields in New York state, USA [33]. Furthermore, the presence of L2 and L1 mitochondrial mtCOI haplotypes within the same cell or individual (i.e., heteroplasmy) has been reported in onion thrips in India [70]. This phenomenon might be explained by paternal leakage during interbreeding of the mentioned lineages; however, other factors can also be involved and require further investigations [70].

### 3.2. Host Plants and Distribution of the Onion Thrips Cryptic Species Complex Based on Their Reproductive Mode

Table 2 lists 56 plant species in which onion thrips were collected and consequently their reproductive mode was determined.

Thelytoky was the most common reproductive mode reported as it was collected in 39 out of the 56 plant species surveyed and it was encountered in all countries sampled. All the works that studied reproductive mode and applied mtCOI identification methods on onion thrips correlate thelytokous reproduction with L2 lineage [14,32,35,36,40,42,78]. In accordance with molecular identification methods presented in Table 1, Table 2 shows that thelytokous populations are the primary lineage recorded in most of the crops where this species is considered a pest of economic importance such as: leek, onion, other *Allium* crops, asparagus, cabbage, cucumber, persimmon, pepper and potato. Conversely, thelytokous populations had minimal or no survival in *Nicotiana* species [10,28,45,49].

**Table 2:** Finding sites and host plants of onion thrips based on their reproductive mode.

Plant Species	Onion Thrips Reproductive Mode	
	Thelytokous	Arrhenotokous
**Amaranthaceae**	-	-
*Amaranthus retroflexus* L.	-	R [87]
*Chenopodium urbicum* L.	-	R [28]
**Amaryllidaceae**	-	-
*Allium ampeloprasum* L.	N/A [46]; R [10,88]	R [10,28,42]
*A. cepa* L.	L [33,43]; N/A [40,79]; R [32]	L [33,43]; N/A [42,79]; R [28,32]
*A. fistulosum* L.	A [77]	A [77]
*A. schoenoprasum* L.	-	R [28]
*A. tuberosum* Rottler ex Spreng.	A [77]	A [77]
*A. × wakegi* Araki	N/A [40]	-
*Allium* sp.	N/A [37,79]	-
**Apiaceae**	-	-
*Coriandrum sativum* L.	N/A [42]	-
**Asparagaceae**	-	-
*Asparagus officinalis* L.	N/A [40]	-
**Asteraceae**	-	-
*Achillea millefolium* L.	-	R [28]
*Arctotheca calendula* (L.) Levyns	A [36]	-
*Chrysanthemum* sp.	A [36]	-
*Cineraria* sp.	A [57]	-
*Emilia coccinea* (Sims) G.Don	R [89]	-
*E. sonchifolia* (L.) DC. ex DC.	R [37]	R [37]
*Erigeron annuus* (L.) Pers.	N/A [42]	-
*Galinsoga parviflora* Cav.	-	R [28]
*Lactuca serriola* L.	-	R [87]
*Santolina chamaecyparissus* L.	N/A [42]	-
*Sonchus oleraceus* (L.) L.	-	R [87]
*Taraxacum officinale* Web	-	R [28]
**Balsaminaceae**	-	-
*Impatiens* sp.	A [36]	R [45]
**Brassicaceae**	-	-
*Brassica oleracea* L.	N/A [32,79]; R [33,38,42,43,75]	N/A [32]; R [33,38,43]
*Brassica* sp.	N/A [79]	-
*Raphanus raphanistrum* subsp. *sativus* (L.) Domin	N/A [37,40,79]	-
*R. raphanistrum* L.	N/A [37,79]	-
**Caprifoliaceae**	-	-
*Lonicera caprifolium* L.	N/A [42]	-
**Cucurbitaceae**	-	-
*Cucumis sativus* L.	R [90]	-
**Ebenaceae**	-	-
*Diospyros kaki* L.f.	A, L [14,39]; N/A [40]	-
**Fabaceae**	-	-
*Medicago sativa* L.	A [36]	-
*Phaseolus vulgaris* L.	R [36,57,70,91]	R [45]
*Trifolium repens* L.	N/A [40]	-
**Fabaceae**	-	-
*Vicia faba* L.	R [14,15,39,40,77]	R [77]
*V. sativa* subsp. *nigra* (L.) Ehrh.	A [14,39]	-
**Lamiaceae**	-	-
*Glechoma hederacea* L.	-	R [28]
*Lamium album* L.	-	N/A [28]
*Lamium* spp.	-	R [28]
**Malvaceae**	-	-
*Gossypium hirsutum* L.	R [92]	-
**Papaveraceae**	-	-
*Chelidonium majus* L.	-	R [28]
**Plantaginaceae**	-	-
*Plantago major* L.	-	R [28]
**Poaceae**	-	-
*Bromus inermis* Leyss.	-	R [28]
*Elymus repens* (L.) Gould	-	R [28]
*Poa annua* L.	-	R [28]
*Secale cereale* L.	N/A [37,79]	-
**Primulaceae**	-	-
*Cyclamen* sp.	A [57]	-
**Rosaceae**	-	-
*Dasiphora fruticosa* (L.) Rydb.	N/A [42]	-
*Filipendula vulgaris* M.	N/A [42]	-
*Sorbaria sorbifolia* (L.) A.Braun	N/A [42]	-
**Rutaceae**	-	-
*Citrus unshiu* (Yu.Tanaka ex Swingle) Marcow	N/A [40]	-
**Solanaceae**	-	-
*Capsicum* sp.	A [57]	-
*Datura stramonium* L.	R [10,45]	R [10,28,45,87]
*Nicotiana tabacum* L.	-	A [10,46]; R [10,28,42,44,87]
*Solanum nigrum* L.	A [36]	R [28,87]
*S. tuberosum* L.	A [36]; N/A [40]	R [28]

Note: Developmental stage in which onion thrips individuals were collected from the plant species under field conditions and then used to determine reproductive mode. A = Adult; L = Larvae; N/A = Not available. R = onion thrips individuals were reared for one or more generations under controlled environmental conditions in the given plant species. - = There are no data.

Arrhenotokous onion thrips were collected and/or reared on 29 plant species in samples collected from Bulgaria [46], Greece [10], Hungary [42], Japan [77], Poland [28], and USA [43]. Arrhenotokous populations were found thriving in most of the *Allium* crops surveyed. These populations are also reported in cabbage crops in the USA. Besides, arrhenotokous lineages were collected in a variety of weed species and solanaceous crops located in central and east Europe [10,28,42,46,87].

Furthermore, Table 1 and Table 2 show that in several entries either only adults were reported, or no information was provided regarding the developmental stage of the individuals collected from the plant species in the field. Hence, it is difficult to establish whether these plant species serve only as a finding site or whether they are true host plants for that particular tested lineage.

In some regions, the presence of arrhenotokous individuals has been reported since only few decades ago; for example, the first confirmed arrhenotokous onion thrips populations were reported in Japan in 1989 [93]. From this point, arrhenotokous populations have spread in this country and now they are often collected in *Allium* species [40,77,94]. These events suggest the current movement of onion thrips populations across countries caused probably by the international trade of agricultural goods; as onion thrips is one of the most commonly intercepted thrips species at ports of entry [9,20].

L1 and T lineages are both generally associated with the arrhenotokous reproductive mode [34,35,38,40,42,78]; as a result, these two lineages cannot be distinguished solely by their reproductive mode. Zawirska [28], found within the arrhenotokous type two strains; one specialised in feeding on tobacco and the other feeding on *Lamium* spp. The author reported that arrhenotokous tobacco-associated strain was able to feed and breed on all plant species tested (i.e., *Nicotiana tabacum, Lamium* spp., *Allium cepa*, *Allium ampeloprasum*, *Allium schoenoprasum*, *Datura stramonium*, *Achillea millefolium*, *Galinsoga parviflora*, *Taraxacum officinale, Solanum nigrum*, *Solanum tuberosum*, *Elymus repens*, *Bromus inermis*, *Poa annua, Chelidonium majus, Chenopodium urbicum*, *Glechoma hederacea* and *Plantago major*). On the contrary, the arrhenotokous *Lamium* species associated strain was able to grow and develop in all plants tested, except tobacco. This host preference information suggest that these two strains might be the tobacco-associated (T) and leek-associated 1 (L1) lineages, respectively, later reported by Brunner et al. [27].

Furthermore, arrhenotokous tobacco-associated populations have been collected from weeds and other crops surrounding tobacco fields; this lineage has also been successfully reared under laboratory conditions using multiple plant species, albeit not identified using molecular identification methods [10,87]. Chatzivassiliou et al. [87], confirmed that arrhenotokous onion thrips breeding in tobacco plants were also able to thrive in weeds surrounding tobacco fields such as *Amaranthus retroflexus*, *Lactuca serriola*, *Sonchus oleraceus*, *Datura stramonium* and *Solanum nigrum*. Their results indicate that tobacco, *D. stramonium*, *L. serriola* and *S. nigrum* were the most suitable host plants for this lineage because the highest oviposition rate, under choice and non-choice conditions, and larval survival rate was observed in these species [87]. The strong preference of the T lineage for tobacco and other solanaceous plants, combined with the ancient origin of this lineage (approximately 28 million years ago) [27], might suggest that this lineage evolved feeding and developing in solanaceous plants native to the presumed centre of origin of onion thrips (eastern Mediterranean region) and later moved to tobacco crop when this plant was introduced to the Palearctic region around 400 years ago [27,95].

**Figure 2:**
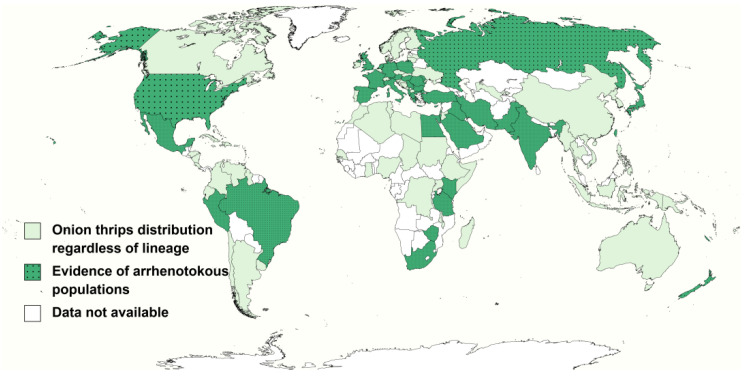
Potential distribution of arrhenotokous onion thrips.

Moreover, there is indirect evidence of arrhenotokous and/or rarely reported deuterotokous onion thrips populations occurring in other parts of the world as the presence of male onion thrips is described in 33 countries and territories. Therefore, their existence is revealed in 39 countries and regions when combining the information from these records and distribution information gathered from Table 1 and Table 2 (Figure 2).

These countries or regions are located in Africa: Egypt [21,23], Kenya [81], South Africa [9], Tanzania [72] and Zimbabwe [9]; America: Brazil [96]; Guadeloupe [97], Martinique [97], Mexico [98], Peru [74] and USA [38,43]; Asia: India [70,99]; Iraq [100], Iran [30], Israel [40,100], Japan [40,94], Pakistan [31], Saudi Arabia [100] and Taiwan island [72]; Europe: Albania [101], Austria [102], Bulgaria [46,103], Cyprus [104], France [105], Germany [106], Greece [10,48], Hungary [3,42], Italy [88], Moldova [107], Netherlands [40,108], Poland [28], Romania [109], Russia [110], Serbia [67], Spain [111], Turkey [112] and United Kingdom [24,113]; Oceania: Hawaii Islands [89], New Caledonia [114], New Zealand [68,115].

Although, several authors mention the predominance of thelytokous populations in most of the countries; these reports are not accompanied by the list of plant species surveyed and/or the methodology followed to reach these conclusions. Additionally, there is plenty of published information reporting that just female adults were collected during thrips surveys [116] or at most a few male adult individuals were collected even in areas with known distribution of the arrhenotokous lineages [3,21,112]. Nevertheless, it is not desirable to relate the presence of only females during the collection events to the predominance of thelytokous populations or absence of arrhenotokous populations since this can be influenced by the sampling methodology and the existence of female biased sex ratio among arrhenotokous populations [34,35,93]. So, the low proportion of males in sympatric populations of onion thrips lineages might go undetected during sampling, especially with low sample size, and consequently an incorrect conclusion is drawn based on the samples only containing females.

The presence of male onion thrips in the sample does not mean that every female individual collected is arrhenotokous, as sympatric populations of the L1 and L2 lineages might be quite common and the presence of the L2 lineage among the females could only be revealed by further investigation. Therefore, the use of molecular identification methods [27,42], rearing methods [60] and morphological identification methods of immature stages [117,118] will help clarify host relations in this cryptic species complex.

Despite the fact that the majority of published information relates thelytoky to the L2 lineage and arrhenotoky to L1 and T lineages, there is evidence indicating that reproductive mode among the onion thrips cryptic species complex might not be a fixed phenotype [15,33,77]. Sogo et al. [77], found arrhenotokous and thelytokous *T. tabaci* populations coexisting in *A. tuberosum* and *A*. *fistulosum* crops in Japan; most of the virgin females phenotyped as arrhenotokous and thelytokous were grouped within the L1 and L2 clades, respectively. However, two virgin females presenting arrhenotokous reproductive mode were grouped within the L2 lineage (generally associated with thelytoky) based on differences of the maternally inherited mtCOI genetic marker. Similar findings were reported by Aizawa et al. [15], as few individuals phenotyped as arrhenotokous had mtCOI sequences that placed them within the L2 lineage [15].

Additionally, the occurrence of deuterotokous reproductive mode was reported twice; one occasion, from few individuals, identified within the L1 and L2 lineages, collected from onion crops in the USA and another time, from a small number of specimens, originally phenotyped as arrhenotokous, collected in Hungary [33,43,44]. Deuterotoky occurring within the order Thysanoptera only has been described in onion thrips and *Apterothrips apteris* (Daniel); in the case of onion thrips, this phenomenon requires additional studies as several hypotheses have been presented regarding its occurrence [43,44].

### 3.3. Host Plant Range of Onion Thrips Regardless of Lineage

Table 3 lists 391 host plants of *T. tabaci*, regardless of lineage; this list includes a wide variety of cereal, legume, fruit, tuber, vegetable, spice, fibre, medicinal, oil, recreational, ornamental and forestry crops, wild plant species, and weeds. These host plants are placed into 64 different botanical families, with the largest numbers of them belonging to Asteraceae (88 plant species), Fabaceae (45), Brassicaceae (36), Poaceae (22) and Solanaceae (18). Notably, adults and larvae were collected from several plant organs, usually vegetative parts [43,58] and inflorescences [58,117]; but also from bulbs [8], corms [8], shoots [1] and fruits [1].

**Table 3:** Confirmed host plants of onion thrips.

Plant Species	References
**Aizoaceae**	-
*Mesembryanthemum forskahlii* Hochst. ex Boiss.	[23]
**Alstroemeriaceae**	-
*Alstroemeria* sp. ^†,‡,§,¶^	[8]
**Amaranthaceae**	-
*Amaranthus albus* L. ^†^	[89]
*A. caudatus* L. ^†^	[23]
*A. retroflexus* L. ^†,‡^	[7,22,89]
*Atriplex hortensis* L.	[22]
*A. sagittata* Borkh.	[22]
*A. tatarica* L.	[119]
*Beta vulgaris* L. ^†^	[22,120]
*Chenopodium album* L. ^†,‡^	[22,23,121]
*C. murale* L. ^†^	[23]
*C. quinoa* Willd. ^†,‡,§^	[122]
*C. urbicum* L. ^†^	[28]
*Dysphania ambrosioides* (L.) Mosyakin and Clemants ^†^	[23]
*Spinacia oleracea* L. ^†^	[22,120]
**Amaryllidaceae**	-
*Allium ampeloprasum* L. ^†,‡^	[10,21,108]
*A. ascalonicum* L.	[123]
*A. cepa* L. ^†,‡^	[21,38,43]
*A. fistulosum* L. ^‡^	[94,124,125]
*A. oleraceum* L.	[22]
*A. sativum* L. ^†,‡^	[22,111,124]
*A. schoenoprasum* L. ^‡^	[28]
*Allium* sp.	[9]
*Narcissus* sp.	[126]
**Apiaceae**	-
*Ammi majus* L. ^†^	[21,23,80]
*Anethum graveolens* L.	[112]
*Anthriscus sylvestris* (L.) Hoffm.	[118]
*Apium graveolens* L. ^†^	[120]
*Conium maculatum* L. ^†^	[52]
*Daucus carota* L. ^†^	[89]
*D. sativus* Roehl.	[22]
*Daucus* sp.	[112]
*Petroselinum crispum* (Mill.) Fuss	[22,127]
**Apocynaceae**	-
*Asclepias syriaca* L.	[17,52]
**Araceae**	-
*Arum* sp. ^†,§^	[113,127]
**Araliaceae**	-
*Polyscias guilfoylei* L.H.Bailey	[89]
*Schefflera* sp.	[8]
**Asparagaceae**	-
*Asparagus officinalis* L.	[124,128]
*Asparagus* sp.	[24]
*Hyacinthus* sp.	[129]
*Yucca* sp.	[89]
**Asteraceae**	-
*Acanthospermum australe* (Loefl.) Kuntze	[89]
*Achillea clypeolata* Sm.	[22]
*A. millefolium* L. ^†^	[22,24,28]
*A. nobilis* L.	[22]
*A. ptarmica* L.	[24]
*A. salicifolia* Besser	[118]
*Achillea* spp.	[130]
*Ageratum conyzoides* (L.) L. ^†^	[23,89,128]
*Ambrosia artemisiifolia* L. ^†^	[7,17,119]
*A. maritima* L.	[21]
*Anthemis arvensis* L. ^†^	[7,22,111]
*A. melampodina* Delile	[21,23]
*A. retusa* Delile	[23]
*Arctium minus* (Hill) Bernh. ^†,‡^	[52]
*Arctotheca calendula* (L.) Levyns ^†^	[80]
*Artemisia princeps* Pamp.	[125]
*A. vulgaris* L. ^†^	[24]
*Aster* spp. ^†^	[24]
*Bidens pilosa* L. ^†^	[80,89,128]
*Brachyscome iberidifolia* Benth. ^†^	[131]
*Calendula arvensis* (Vaill.) L. ^†^	[132]
*C. officinalis* L. ^†^	[59]
*Callistephus chinensis* (L.) Nees ^†^	[128]
*Carduus acanthoides* L. ^†^	[22,133]
*C. pycnocephalus* L. ^†^	[22]
*Carlina vulgaris* L.	[118]
*Centaurea calcitrapa* L. ^†^	[23]
*C. cyanus* L. ^†^	[22]
*Chrysanthemum* spp. ^†,‡,§^	[24,59,89]
*Cichorium endivia* L. ^†^	[23,120]
*C. intybus* L. ^†,‡^	[23,120,121]
*Cineraria* sp. ^†^	[24,113]
*Cirsium arvense* (L.) Scop. ^†^	[22,52]
*C. syriacum* (L.) Gaertn.	[21,23]
*Cota tinctoria* (L.) J.Gay ^†^	[22]
*Cyanthillium cinereum* (L.) H.Rob.	[89]
*Dahlia pinnata* Cav.	[22]
*Dahlia* sp. ^†,§^	[8]
*Emilia coccinea* (Sims) G.Don ^†^	[89,128]
*E. sonchifolia* (L.) DC. Ex DC. ^†,§,¶^	[37,134]
*Emilia* spp. ^†^	[128]
*Erigeron annuus* (L.) Pers.	[135]
*Erigeron bonariensis* L. ^†^	[23]
*E. canadensis* L. ^†,‡^	[22,89,128]
*Eupatorium cannabinum* L. ^†^	[22]
*Galinsoga parviflora* Cav. ^†^	[7,17,119]
*Helianthus annuus* L. ^†,‡^	[22]
*Helminthotheca echioides* (L.) Holub ^†^	[133]
*Hypochaeris radicata* L.	[24]
*Inula candida* (L.) Cass.	[22]
*I. helenium* L. ^†^	[22]
*I. salicifolia* Gueldenst.	[136]
*Jacobaea maritima* (L.) Pelser and Meijden ^†^	[22]
*Lactuca sativa* L. ^†,§^	[22,121,128]
*L. serriola* L. ^†,‡^	[64,87]
*Laphangium luteoalbum* (L.) Tzv.	[23]
*Launaea nudicaulis* (L.) Hook.f.	[23]
*Leucanthemum vulgare* Lam. ^†^	[8,22]
*Matricaria chamomilla* L. ^†^	[22,121,133]
*Matricaria* spp.	[24]
*Pluchea indica* (L.) Less.	[89]
*Pulicaria arabica* (L.) Cass.	[23]
*Santolina chamaecyparissus* L.	[137]
*Senecio cruentus* (L’Hér.) DC. ^†,§^	[12]
*S. glaucus* subsp. *coronopifolius* C. Alex.	[21]
*S. pogonias* Cabrera	[121]
*S. umbrosus* Waldst. and Kit.	[118]
*S. viscosus* L.	[24]
*S. vulgaris* L. ^†,‡^	[119,135]
*Senecio* spp. ^†^	[24,130]
*Silybum marianum* (L.) Gaertn. ^†^	[23]
*Solidago canadensis* L. ^†^	[52]
*S. gigantea* Aiton	[118]
*S. virgaurea* L.	[118]
*Solidago* spp. ^†^	[24]
*Sonchus asper* (L.) Hill ^†,‡^	[138]
*S. oleraceus* (L.) L. ^†,‡^	[23,58,80]
*Tanacetum cinerariifolium* (Trevir.) Sch.Bip. ^†^	[21,22]
*T. vulgare* L.	[118]
*Taraxacum officinale* (L.) Weber ex F.H.Wigg. ^†,‡^	[7,28,52]
*Tithonia rotundifolia* (Mill.) S.F.Blake ^†^	[89]
*Tragopogon dubius* Scop. ^‡^	[64]
*T. porrifolius* L. ^†^	[22]
*Tripleurospermum indorum* (L.) Sch.Bip. ^†^	[22]
*T. maritmum* (L.) Koch	[119]
*Verbesina encelioides* (Cav.) Benth. and Hook.f. ex A.Gray ^†^	[55]
*Xanthium strumarium* L. ^†^	[80]
*Zinnia elegans* L. ^†^	[22]
**Balsaminaceae**	**-**
*Impatiens* sp. ^†,‡,§,¶^	[45]
**Boraginaceae**	-
*Anchusa azurea* Mill. ^†^	[111]
*Bothriospermum tenellum* (Hornem.) Fisch. and Mey.	[89]
*Echium plantagineum* L.	[58,80,133]
*Heliotropium europaeum* L. ^†^	[22]
**Brassicaceae**	-
*Alyssum* spp.	[26]
*Armoracia rusticana* P.Gaertn., B.Mey. and Scherb.	[139]
*Barbarea vulgaris* R.Br. ^†^	[52]
*Berteroa incana* (L.) DC.	[22]
*Biscutella auriculata* L.	[111]
*Brassica cretica* Lam.	[22]
*B. juncea* (L.) Czern.	[140]
*B. napus* L. ^†^	[80]
*B. nigra* (L.) K.Koch	[89]
*B. oleracea* L. ^†^	[21,38,60]
*B. rapa* L. ^†^	[22,133]
*Brassica* sp.	[125]
*Capsella bursa-pastoris* (L.) Medik. ^†^	[52,80,121]
*Cochlearia* sp.	[24]
*Descurainia sophia* (L.) Webb ex Prantl ^‡^	[64]
*Diplotaxis* sp.	[111]
*Erophila verna* (L.) DC.	[22]
*Eruca vesicaria* (L.) Cav.	[21,23,121]
*Erysimum* sp.	[24]
*Lepidium bonariense* L.	[80]
*L. didymum* L. ^†^	[89]
*L. draba* L. ^†^	[22,135]
*L. latifolium* L.	[22]
*L. virginicum* L. ^†^	[52]
*Lobularia* spp.	[26]
*Raphanus raphanistrum* subsp. *sativus* (L.) Domin	[23,89,133]
*R. raphanistrum* L. ^†^	[52]
*Raphanus* sp.	[141]
*Rapistrum rugosum* (L.) All.	[58,80,133]
*Sinapis alba* L.	[112]
*S. arvensis* L. ^†,‡^	[22,52]
*Sisymbrium irio* L.	[21,23,121]
*S. officinale* (L.) Scop.	[22]
*Sisymbrium* sp.	[111]
*Thlaspi arvense* L.	[52]
**Bromeliaceae**	-
*Ananas comosus* (L.) Merr. ^†^	[89,128]
**Cactaceae**	-
*Opuntia* sp. ^†^	[24]
**Calceolariaceae**	-
*Calceolaria* sp. ^†^	[127]
**Campanulaceae**	-
*Campanula* sp. ^†^	[24]
**Caricaceae**	-
*Carica papaya* L. ^†^	[120,142]
**Caryophyllaceae**	-
*Dianthus barbatus* L.	[8]
*D. campestris* M.Bieb.	[22]
*D. caryophyllus* L.	[21,59]
*D. corymbosus* Sm.	[22]
*Dianthus* spp. ^†^	[8,24,89]
*Spergularia rubra* (L.) J.Pr. and C.Pr.	[24]
*Stellaria media* (L.) Vill. ^†^	[7,51,119]
**Commelinaceae**	-
*Commelina* sp.	[120]
*Murdannia nudiflora* (L.) Brenan	[120]
*Tradescantia* sp. ^†^	[24]
**Convolvulaceae**	-
*Calystegia sepium* (L.) R. Br. ^†^	[52]
*Convolvulus arvensis* L. ^†,‡^	[7,21,121]
*Ipomoea batatas* (L.) Lam. ^†^	[22,125]
*I. indica* (Burm.) Merr. ^†^	[128]
*Operculina aequisepala* (Domin)	[80]
**Crassulaceae**	-
*Sedum acre* L.	[118]
*Sedum* sp. ^†^	[24]
**Cucurbitaceae**	-
*Citrullus lanatus* (Thunb.) Matsum. and Nakai ^†^	[21,22]
*Cucumis melo* L. ^†^	[22]
*C. sativus* L. ^†,§^	[8,90,125]
*Cucurbita maxima* Duchesne ^†^	[22]
*C. moschata* Duchesne ^†^	[21,22]
*C. pepo* L. ^†^	[21,22]
**Ebenaceae**	-
*Diospyros kaki* L.f.	[14]
**Ericaceae**	-
*Ledum palustre* L.	[118]
**Euphorbiaceae**	-
*Chrozophora tinctoria* (L.) A.Juss.	[21]
*Euphorbia hirta* L.	[89]
*Ricinus communis* L. ^†,‡^	[23]
**Fabaceae**	-
*Anthyllis vulneraria* L.	[118]
*Anthyllis* sp.	[24]
*Arachis hypogaea* L. ^†^	[22]
*Astragalus cicer* L.	[22]
*Bauhinia* sp.	[23]
*Cajanus cajan* (L.) Millsp. ^†^	[89]
*Cicer arietinum* L. ^†^	[117]
*Crotalaria juncea* L. ^†^	[89]
*C. saltiana* Andrews	[89]
*C. spectabilis* Roth ^†^	[89]
*Cytisus nigricans* L.	[135]
*Delonix regia* (Boj.ex Hook.) Raf.	[21]
*Galega officinalis* L.	[133]
*Glycine max* (L.) Merr. ^†,§^	[22,143]
*Hippocrepis emerus* (L.) Lassen	[22]
*Lathyrus* sp.	[24]
*Lens culinaris* Medik ^†^	[21]
*Lotus corniculatus* L.	[22]
*L. tenuis* Waldst. and Kit.	[133]
*Lotus* sp.	[24]
*Lupinus angustifolius* L. ^†^	[144]
*Lupinus* sp. ^†^	[89]
*Medicago lupulina* L. ^†^	[22,52]
*M. polymorpha* var. *vulgaris* (Benth.) Shinners	[80]
*M. sativa* L.	[21,80,89]
*Melilotus albus* Medik.	[118]
*M. indicus* (L.) All. ^†^	[21,23]
*M. messanensis* (L.) All.	[23]
*M. officinalis* (L.) Pall. ^†^	[22,119]
*Mimosa pudica* L.	[89]
*Ornithopus compressus L.*	[22]
*Phaseolus vulgaris* L. ^†,¶^	[21,22,121]
*Pisum sativum* L. ^†,§^	[89,117,128]
*P. sativum* subsp. *elatius* (M.Bieb.) Asch. and Graebn.	[22]
*Senna occidentalis* (L.) Link ^†^	[89]
*Trifolium alexandrinum* L.	[21,112]
*T. pratense* L.	[22,112]
*T. repens* L. ^†^	[7,22,133]
*Trifolium resupinatum* L.	[23]
*Trigonella foenum-graecum* L.	[21]
*T. laciniata* L.	[23]
*Vicia faba* L. ^†,§,¶^	[21,77,80]
*V. sativa* L. ^‡^	[80,121]
*Vigna mungo* (L.) Hepper ^†^	[145]
*V. unguiculata* subsp. *unguiculata* (L.) Walp. ^†^	[89]
**Gentianaceae**	-
*Eustoma russellianum* (Hook.) G.Don ^†,‡^	[146]
**Geraniaceae**	-
*Erodium cicutarium* (L.) L’Hér. ^‡^	[22]
*Geranium pratense* L.	[118]
*G. pusillum* L.	[22]
*G. pyrenaicum* Burm.f.	[22]
**Gesneriaceae**	-
*Saintpaulia brevipilosa* B.L.Burtt	[131]
**Hypericaceae**	-
*Hypericum perforatum* L.	[22]
**Iridaceae**	-
*Freesia* sp.	[8]
*Gladiolus* spp. ^†^	[8]
**Juncaginaceae**	-
*Triglochin maritima* L.	[24]
**Lamiaceae**	-
*Glechoma hederacea* L.	[22,28]
*Lamium album* L.	[28]
*L. amplexicaule* L. ^†^	[7,23,121]
*L. purpureum* L. ^†^	[22,52]
*Lamium* spp.	[28]
*Nepeta cataria* L. ^†^	[52]
*Nepeta* spp.	[24]
*Origanum vulgare* L.	[121]
*Rosmarinus* spp.	[26]
*Salvia* sp. ^†^	[24]
*Teucrium scorodonia* L.	[24]
**Linaceae**	-
*Linum usitatissimum* L.	[147]
**Lythraceae**	-
*Cuphea hyssopifolia* Kunth	[89]
**Malvaceae**	-
*Abutilon theophrasti* Medik.	[22]
*Alcea rosea* L.	[21,22]
*Althaea officinalis* L. ^†^	[22]
*Althaea* sp. ^†,§^	[24]
*Gossypium arboreum* L.	[22]
*Gossypium barbadense* L. ^†^	[22]
*G. hirsutum* L. ^†^	[22,80]
*Gossypium* spp.	[21]
*Malva neglecta* Wallr. ^†^	[22,52,64]
*M. parviflora* L. ^†^	[121]
*M. sylvestris* L. ^†^	[22]
*Malva* sp. ^†^	[89]
*Sphaeralcea miniata* (Cav.) Spach	[121]
*Tilia cordata* Mill.	[22]
*T. tomentosa* Moench	[22]
*Waltheria indica* L.	[89]
**Moraceae**	-
*Ficus carica* L.	[148]
*Morus alba* L.	[21,22]
*M. nigra* L.	[22]
**Myrtaceae**	-
*Eucalyptus* spp.	[26]
**Nyctaginaceae**	-
*Boerhavia glabrata* Blume	[80]
**Onagraceae**	-
*Oenothera biennis* L. ^†^	[52]
**Oxalidaceae**	-
*Oxalis corniculata* L. ^†^	[23]
*O. debilis* Kunth	[89]
*O. stricta* L. ^†^	[52]
*Oxalis* sp. ^†^	[24]
**Papaveraceae**	-
*Chelidonium majus* L.	[28]
*Fumaria capreolata* L.	[80]
*Papaver rhoeas* L. ^†^	[111]
**Phytolaccaceae**	-
*Phytolacca acinosa* Roxb.	[89]
**Plantaginaceae**	-
*Digitalis* sp. ^†^	[24]
*Linaria vulgaris* Mill.	[22,112]
*Plantago lanceolata* L. ^†,‡^	[52,121]
*P. major* L. ^†^	[28]
*P. maritima* L.	[24]
*Veronica austriaca* L.	[22]
*V. persica* Poir. ^†^	[121]
**Plumbaginaceae**	-
*Armeria* sp.	[24]
**Poaceae**	-
*Avena fatua* L. ^†^	[80]
*A. sativa* L.	[149]
*Avena* sp.	[52]
*Bromus inermis* Leyss. ^‡^	[28]
*Cenchrus americanus* (L.) Morr.	[22]
*Dactylis glomerata* L.	[111]
*Digitaria sanguinalis* (L.) Scop. ^†^	[22,89]
*D. violascens* Link	[89]
*Eleusine indica* (L.) Gaertn. ^†,‡^	[89]
*Elymus repens* (L.) Gould	[28]
*Hordeum maritimum* With.	[21,23]
*H. vulgare* L.	[21,60,149]
*Lolium* sp.	[52]
*Phleum pratense* L.	[149]
*Poa annua* L. ^†^	[28]
*Polypogon monspeliensis* (L.) Desf	[23]
*P. viridis* (Gouan) Breistr.	[23]
*Secale cereale* L.	[150]
*Setaria verticillata* (L.) P.Beauv.	[22]
*S. viridis* (L.) P.Beauv. ^†,‡^	[22]
*Sorghum halepense* (L.) Pers.	[22]
*Triticum aestivum* L. ^‡^	[58,149,150]
**Polygonaceae**	-
*Persicaria maculosa* Gray ^†^	[22]
*P. orientalis* (L.) Spach	[80]
*Polygonum aviculare* L. ^†^	[80]
*Rumex crispus* L. ^†,‡^	[58,80]
*R. dentatus* L.	[23]
*R. obtusifolius* L.	[119]
**Portulacaceae**	-
*Portulaca oleracea* L. ^†,‡,§^	[89]
**Primulaceae**	-
*Cyclamen coum* Mill.	[22]
*Cyclamen* spp. ^†^	[8,127]
**Ranunculaceae**	-
*Helleborus odorus* Waldst. and Kit.	[22]
*Ranunculus arvensis* L. ^†^	[22]
*R. polyanthemos* L.	[22]
**Resedaceae**	-
*Reseda* spp.	[26]
**Rosaceae**	-
*Fragaria × ananassa* (Duchesne ex Weston) Duchesne ex Rozier	[151]
*Malus domestica* Borkh.	[22]
*Potentilla alba* L.	[22]
*Prunus avium* (L.) L.	[152]
*P. domestica* L.	[22]
*P. persica* (L.) Batsch	[24]
*Prunus* spp.	[26]
*Pyrus communis* L.	[22]
*Rosa canina* L.	[22]
*R. damascena* Mill.	[22]
*Rosa* sp. ^†,‡,§^	[9,127]
**Rubiaceae**	-
*Galium aparine* L. ^†^	[22]
*G. verum* L. ^†^	[118,119]
*Galium* sp. ^†^	[24]
*Richardsonia scabra* L. ^†^	[89,128]
**Rutaceae**	-
*Citrus unshiu* (Yu.Tanaka ex Swingle) Marcow	[153]
*Citrus* spp.	[154]
**Salicaceae**	-
*Salix mucronata* Thunb.	[21]
*Salix* sp.	[23]
**Scrophulariaceae**	-
*Verbascum lychnitis* L.	[118]
*V. thapsus* L. ^†^	[52]
**Solanaceae**	-
*Alkekengi officinarum* Moench ^†^	[22]
*Atropa bella-donna* L. ^†^	[22]
*Capsicum annuum* L. ^†,‡,§^	[120,155]
*Datura stramonium* L. ^†,‡,§,¶^	[28,45,120]
*Hyoscyamus niger* L. ^†^	[22]
*Lycium barbarum* L.	[22]
*Nicandra physalodes* (L.) Gaertn. ^†^	[89]
*Nicotiana glauca* Graham ^†^	[128]
*N. rustica* L. ^†,‡,§^	[22]
*N. tabacum* L. ^†,§,¶^	[10,28,107]
*Nicotiana* sp. ^†^	[112]
*Petunia hybrida* Vilm ^†,‡,§^	[22]
*Solanum americanum* Mill. ^†^	[89,128]
*S. dulcamara* L. ^†^	[22]
*S. lycopersicum* L. ^†,‡,§^	[21,22,23]
*S. melongena* L. ^†,§^	[22,120,155]
*S. nigrum* L. ^†,‡^	[22,28,87]
*S. tuberosum* L. ^†,‡,§^	[21,28,120]
**Tropaeolaceae**	-
*Tropaeolum majus* L. ^†,§^	[22]
**Urticaceae**	-
*Urtica dioica* L. ^†^	[52]
**Verbenaceae**	-
*Lantana camara* L.	[89]
*Pitraea cuneato-ovata* (Cav.) Caro	[121]
*Stachytarpheta cayennensis* (Rich.) Vahl	[89]
*Verbena bonariensis* L.	[89]
*V. officinalis* L. ^†^	[80]
*V. tenuisecta* Briq.	[58]
**Viburnaceae**	-
*Sambucus ebulus* L.	[22]
**Vitaceae**	-
*Vitis vinifera* L.	[22]
**Zygophyllaceae**	-
*Tribulus terrestris* L. ^†,‡^	[22,80]

Note: Plant species known to host: ^†^ Tomato Spotted Wilt Virus (TSWV) [62,63]; ^‡^ Iris Yellow Spot Virus (IYSV) [11,52,63,64,65]; ^§^ Tomato Yellow Ring Virus (TYRV) [12,63,66]; and ^¶^ Alstroemeria Yellow Spot Virus (AYSV) [13]. - = There are no data.

Table 3 quantifies the highly polyphagous nature of this species complex; even though to our best knowledge, this list of host plants is the most comprehensive list presented to date for this species, the complete host range of the onion thrips cryptic species complex is likely to be even broader since most of the surveys carried out either only involve the identification of adults or are biased towards studying plant species with agricultural importance [87,112]. Likewise, taking into account its global distribution (Figure 1 and Figure 2) [2], the number of botanical families in which this species is reported to breed and the reports of thrips adults being collected from hundreds of plant species at national level [21,22,23,24,89], it is plausible that many more plants will be reported as breeding grounds for onion thrips.

Table 3 also indicates that 187, 44, 25 and 7 of the listed plant species are, at the same time, hosts of the economically important plant orthotospoviruses TSWV, IYSV, TYRV, and AYSV, respectively. These results indicate the potential of these plant species as both virus reservoirs and virus acquisition sites for the larval stages of onion thrips; consequently, the integrated pest management strategies for both virus and vector must prioritise practices aiming the removal of the above-mentioned plant species, timed insecticide application targeting early infestation sources, along with other vector management approaches [17,50].

In addition, every host plant has different prominence to a polyphagous species such as onion thrips and trying to get into details about these relationships will go beyond the scope of this article. Nonetheless, it is important to notice that: (1) some plant species are more suitable as feeding and breeding grounds than others [52,58,80,87]. (2) Other plants have critical importance for the survival of the species during winter or in periods when the preferred host is not available in the environment [6,50,116,149]. (3) Other hosts are so abundant that they represent a readily available food source and breeding site, even if it is not the preferred host plant [6,52]. (4) Finally, insect preference for any host plant might depend on the phenological stage of the plant and/or specific plant organs. For instance, several references found significantly higher adult and larval densities on floral organs than on vegetative parts in every host analysed [58,80].

## 4. Conclusions

The full host range and distribution of the onion thrips cryptic species complex has not been established with certainty to date. Significant efforts have been carried out to elucidate the plant species that allow its survival mainly within agroecosystems; as a result, numerous plant species have been identified as breeding sites for onion thrips at species level. However, the study of the host range at lineage level still only comprises crops plants in which this insect is considered a pest. L2 is the most common lineage as it is being collected from all studied regions and from the majority of plants species sampled, including major crops from different botanical families. L1 lineage has been reported from 16 countries and territories, mainly from onion species. T lineage has only been collected from tobacco and weeds growing near tobacco fields from localised regions. To achieve a more complete state of knowledge, the study of the relationship between onion thrips lineages and host plants must integrate different tools including repetitive sample collections on the target plants, the use of both morphological and genetic identification methods and host preference investigations under controlled environmental conditions.

## Data Availability

Not applicable.

## References

[1] Lewis T., Mound L.A., Nakahara S., Childers C.C., Lewis T. (1997). Major crops infested by thrips with main symptoms and predominant injurious species. Thrips as Crop Pests.

[2] CAB International Thrips Tabaci (Onion Thrips). https://www.cabi.org/isc/datasheet/53746.

[3] Jenser G., Lipcsei S., Szénási A., Hudák K. (2006). Host range of the arrhenotokous populations of *Thrips tabaci* (Thysanoptera: Thripidae). Acta Phytopathol. Entomol. Hung..

[4] Diaz-Montano J., Fuchs M., Nault B.A., Fail J., Shelton A.M. (2011). Onion thrips (Thysanoptera: Thripidae): A global pest of increasing concern in onion. J. Econ. Entomol..

[5] Mound L.A., Marullo R., Virendra K.G. (1996). The Thrips of Central and South America: An Introduction (Insecta: Thysanoptera).

[6] Shelton A.M., North R.C. (1986). Species composition and phenology of Thysanoptera within field crops adjacent to cabbage fields. Environ. Entomol..

[7] Orosz S., Éliás D., Balog E., Tóth F. (2017). Investigation of Thysanoptera populations in Hungarian greenhouses. Acta Univ. Sapientiae Agric. Environ..

[8] Mantel W.P., van de Vrie M. (1988). A contribution to the knowledge of Thysanoptera in ornamental and bulbous crops in the Netherlands. Acta Phytopathol. Entomol. Hung..

[9] Vierbergen G. (2014). Thysanoptera intercepted in the Netherlands on plant products from Ethiopia, with description of two new species of the genus *Thrips*. Zootaxa.

[10] Chatzivassiliou E.K., Peters D., Katis N.I. (2002). The efficiency by which *Thrips tabaci* populations transmit *Tomato spotted wilt virus* depends on their host preference and reproductive strategy. Phytopathology.

[11] Bag S., Schwartz H.F., Cramer C.S., Havey M.J., Pappu H.R. (2015). *Iris yellow spot virus* (Tospovirus: Bunyaviridae): From obscurity to research priority. Mol. Plant Pathol..

[12] Rasoulpour R., Izadpanah K. (2007). Characterisation of cineraria strain of *Tomato yellow ring virus* from Iran. Australas. Plant Pathol..

[13] Hassani-Mehraban A., Dullemans A.M., Verhoeven J.T.J., Roenhorst J.W., Peters D., van der Vlugt R.A.A., Kormelink R. (2019). *Alstroemeria yellow spot virus* (AYSV): A new orthotospovirus species within a growing Eurasian clade. Arch. Virol..

[14] Morishita M. (2008). Pyrethroid-resistant onion thrips, *Thrips tabaci* Lindeman (Thysanoptera: Thripidae), infesting persimmon fruit. Appl. Entomol. Zool..

[15] Aizawa M., Watanabe T., Kumano A., Miyatake T., Sonoda S. (2016). Cypermethrin resistance and reproductive types in onion thrips, *Thrips tabaci* (Thysanoptera: Thripidae). J. Pestic. Sci..

[16] Gill H.K., Garg H., Gill A.K., Gillett-Kaufman J.L., Nault B.A. (2015). Onion thrips (Thysanoptera: Thripidae) biology, ecology, and management in onion production systems. J. Integr. Pest Manag..

[17] Jenser G., Almási A., Kazinczi G., Takács A., Szénási Á., Gáborjányi R. (2009). Ecological background of the epidemics of *Tomato spotted wilt virus* in central Europe. Acta Phytopathol. Entomol. Hung..

[18] Chatzivassiliou E.K. (2008). Management of the spread of *Tomato spotted wilt virus* in tobacco crops with insecticides based on estimates of thrips infestation and virus incidence. Plant Dis..

[19] Gent D.H., du Toit L.J., Fichtner S.F., Mohan S.K., Pappu H.R., Schwartz H.F. (2006). *Iris yellow spot virus*: An emerging threat to onion bulb and seed production. Plant Dis..

[20] Lewis T., Lewis T. (1997). Flight and dispersal. Thrips as Crop Pests.

[21] Ghabn A.A.A.E. (1948). Contribution to the knowledge of the biology of *Thrips tabaci* Lind. in Egypt. Bull. Soc. Entomol. Egypte.

[22] Dimitrov A. (1977). Host plants of *Thrips tabaci* Lind. in Bulgaria. Plant Sci..

[23] Priesner H. (1960). A Monograph of the Thysanoptera of the Egyptian Deserts.

[24] Morison G.D. (1943). Notes on Thysanoptera found on flax (*Linum usitatissimum* L.) in the British isles. Ann. Appl. Biol..

[25] Mound L.A. (2013). Homologies and host-plant specificity: Recurrent problems in the study of thrips. Fla. Entomol..

[26] Marullo R. (2004). Host-plant range and relationships in the Italian thrips fauna. Acta Phytopathol. Entomol. Hung..

[27] Brunner P.C., Chatzivassiliou E.K., Katis N.I., Frey J.E. (2004). Host-associated genetic differentiation in *Thrips tabaci* (Insecta; Thysanoptera), as determined from mtDNA sequence data. Heredity.

[28] Zawirska I. (1976). Untersuchungen über zwei biologische typen von *Thrips tabaci* Lind. (Thysanoptera, Thripidae) in der VR Polen. Arch. Phytopathol. Pflanzenschutz.

[29] Glover R.H., Collins D.W., Walsh K., Boonham N. (2010). Assessment of loci for DNA barcoding in the genus *Thrips* (Thysanoptera:Thripidae). Mol. Ecol. Resour..

[30] Fekrat L., Manzari S., Shishehbor P. (2014). Molecular and morphometric variation of *Thrips tabaci* Lindeman (Thysanoptera: Thripidae) populations on onion and tobacco in Iran. J. Agric. Sci. Technol..

[31] Iftikhar R., Ashfaq M., Rasool A., Hebert P.D.N. (2016). DNA barcode analysis of thrips (Thysanoptera) diversity in Pakistan reveals cryptic species complexes. PLoS ONE.

[32] Li X.W., Wang P., Fail J., Shelton A.M. (2015). Detection of gene flow from sexual to asexual lineages in *Thrips tabaci* (Thysanoptera: Thripidae). PLoS ONE.

[33] Jacobson A.L., Nault B.A., Vargo E.L., Kennedy G.G. (2016). Restricted gene flow among lineages of *Thrips tabaci* supports genetic divergence among cryptic species groups. PLoS ONE.

[34] Kobayashi K., Yoshimura J., Hasegawa E. (2013). Coexistence of sexual individuals and genetically isolated asexual counterparts in a thrips. Sci. Rep..

[35] Kobayashi K., Hasegawa E. (2016). A female-biased sex ratio reduces the twofold cost of sex. Sci. Rep..

[36] Westmore G.C., Poke F.S., Allen G.R., Wilson C.R. (2013). Genetic and host-associated differentiation within *Thrips tabaci* Lindeman (Thysanoptera: Thripidae) and its links to *Tomato spotted wilt virus*-vector competence. Heredity.

[37] Jacobson A.L., Kennedy G.G. (2013). Specific insect-virus interactions are responsible for variation in competency of different *Thrips tabaci* isolines to transmit different *Tomato spotted wilt virus* isolates. PLoS ONE.

[38] Li X.W., Fail J., Wang P., Feng J., Shelton A.M. (2014). Performance of arrhenotokous and thelytokous *Thrips tabaci* (Thysanoptera: Thripidae) on onion and cabbage and its implications on evolution and pest management. J. Econ. Entomol..

[39] Toda S., Morishita M. (2009). Identification of three point mutations on the sodium channel gene in pyrethroid-resistant *Thrips tabaci* (Thysanoptera: Thripidae). J. Econ. Entomol..

[40] Toda S., Murai T. (2007). Phylogenetic analysis based on mitochondrial COI gene sequences in *Thrips tabaci* Lindeman (Thysanoptera: Thripidae) in relation to reproductive forms and geographic distribution. Appl. Entomol. Zool..

[41] Kobayashi K., Hasegawa E. (2012). Discrimination of reproductive forms of *Thrips tabaci* (Thysanoptera: Thripidae) by PCR with sequence specific primers. J. Econ. Entomol..

[42] Farkas P., György Z., Tóth A., Sojnóczki A., Fail J. (2020). A simple molecular identification method of the *Thrips tabaci* (Thysanoptera: Thripidae) cryptic species complex. Bull. Entomol. Res..

[43] Nault B.A., Shelton A.M., Gangloff-kaufmann J.L., Clark M.E., Werren J.L., Cabrera-la Rosa J.C., Kennedy G.G. (2006). Reproductive modes in onion thrips (Thysanoptera: Thripidae) populations from New York onion fields. Environ. Entomol..

[44] Woldemelak W.A. (2020). The existence of deuterotokous reproduction mode in the *T. tabaci* (Thysanoptera: Thripidae) cryptic species complex. J. Hortic. Res..

[45] Wijkamp I. (1995). Distinct levels of specificity in thrips transmission of tospoviruses. Phytopathology.

[46] Karadjova O., Krumov V. (2008). TSWV transmission efficiency of an arrhenotokous and a thelytokous population of *Thrips tabaci*. Acta Phytopathol. Entomol. Hung..

[47] Hristova D., Karadjova O., Yankulova M., Heinze C., Adam G. (2001). A survey of tospoviruses in Bulgaria. J. Phytopathol..

[48] Chatzivassiliou E.K., Nagata T., Katis N.I., Peters D. (1999). Transmission of *Tomato spotted wilt tospovirus* by *Thrips tabaci* populations originating from leek. Plant Pathol..

[49] Inoue T., Murai T., Natsuaki T. (2010). An effective system for detecting *Iris yellow spot virus* transmission by *Thrips tabaci*. Plant Pathol..

[50] Jenser G., Gaborjanyi R., Szenasi A., Almasi A., Grasselli M. (2003). Significance of hibernated *Thrips tabaci* Lindeman (Thysan., Thripidae) adults in the epidemic of *Tomato spotted wilt virus*. J. Appl. Entomol..

[51] Jenser G., Vierbergen B., Szénási A. (2007). Thysanoptera larvae living on chickweed (*Stellaria media* Linnaeus) under continental climatic conditions. Acta Phytopathol. Entomol. Hung..

[52] Smith E.A., Ditommaso A., Fuchs M., Shelton A.M., Nault B.A. (2011). Weed hosts for onion thrips (Thysanoptera: Thripidae) and their potential role in the epidemiology of *Iris yellow spot virus* in an onion ecosystem. Environ. Entomol..

[53] Trdan S., Žnidarčič D., Valič N., Rozman L., Vidrih M. (2006). Intercropping against onion thrips, *Thrips tabaci* Lindeman (Thysanoptera: Thripidae) in onion production: On the suitability of orchard grass, lacy phacelia, and buckwheat as alternatives for white clover. J. Plant Dis. Prot..

[54] Buckland K.R., Alston D.G., Reeve J.R., Nischwitz C., Drost D. (2017). Trap crops in onion to reduce onion thrips and *Iris yellow spot virus*. Southwest. Entomol..

[55] Calvert F., Hollingsworth R.G., Wall M., Follett P.A. (2019). Survey of flowering plants in Hawaii as potential banker plants of anthocorid predators for thrips control. J. Asia Pac. Entomol..

[56] Ren X., Wu S., Xing Z., Gao Y., Cai W., Lei Z. (2020). Abundances of thrips on plants in vegetative and flowering stages are related to plant volatiles. J. Appl. Entomol..

[57] Tavella L., Tedeschi R., Mason G., Roggero P., Marullo R., Mound L.A. (2002). Efficiency of north-western Italian thrips populations in transmitting tospoviruses. Proceedings of the 7th International Symposium on Thysanoptera.

[58] Milne M., Walter G.H. (1998). Host species and plant part specificity of the polyphagous onion thrips, *Thrips tabaci* Lindeman (Thysanoptera: Thripidae), in an Australian cotton-growing area. Aust. J. Entomol..

[59] Zamar M.I., Neder L.E., Linares M.A., Hamity V.C., Contreras E.F., Gómez G. (2014). Tisanópteros (Insecta) asociados a plantas ornamentales de Jujuy (Argentina). Rev. Agron. Noroeste Argent..

[60] de Borbón C., Gracia O., De Santis L. (1999). Survey of Thysanoptera occurring on vegetable crops as potential tospovirus vectors in Mendoza, Argentina. Rev. Soc. Entomol. Argen..

[61] Teulon D.A.J., Groninger J.W., Cameron E.A. (1994). Distribution and host plant associations of *Taeniothrips inconsequens* (Thysanoptera: Thripidae). Environ. Entomol..

[62] Parrella G., Gognalons P., Gebre-Selassiè K., Vovlas C., Marchoux G. (2003). An update of the host range of *Tomato spotted wilt virus*. J. Plant Pathol..

[63] Sastry K.S., Mandal B., Hammond J., Scott S.W., Briddon R.W. (2019). Encyclopedia of Plant Viruses and Viroids.

[64] Szostek S., Schwartz H.F. (2015). Overwintering sites of *Iris yellow spot virus* and *Thrips tabaci* (Thysanoptera: Thripidae) in Colorado. Southwest. Entomol..

[65] Schwartz H.F., Gent D.H., Fichtner S.M., Otto K., Boateng C.O., Szostek S., Cranshaw W.S., Mahaffey L.A. (2014). *Thrips tabaci* (Thysanoptera: Thripidae) and *Iris yellow spot virus* associated with onion transplants, onion volunteers, and weeds in Colorado. Southwest. Entomol..

[66] Hassani-Mehraban A., Saaijer J., Peters D., Goldbach R., Kormelink R. (2005). A new tomato-infecting tospovirus from Iran. Phytopathology.

[67] Cvrković T., Jović J., Mitrović M., Krstić O., Toševski I., Marisavljevic D. (2012). Genetic variability in *Thrips tabaci* (Insecta: Thysanoptera) living on vegetables in Serbia. Proceedings of the International Symposium on Current Trends in Plant Protection.

[68] Schmidt K. (2007). *Thrips obscuratus* in New Zealand Vineyards: Its Biology and Effects on Botrytis Cinerea Infection. Ph.D. Thesis.

[69] Li X., Zhang Z., Zhang J., Huang J., Wang L., Li Y., Hafeez M., Lu Y. (2020). Population genetic diversity and structure of *Thrips tabaci* (Thysanoptera: Thripidae) on *Allium* hosts in China, inferred from mitochondrial COI gene sequences. J. Econ. Entomol..

[70] Gawande S.J., Anandhan S., Ingle A.A., Jacobson A., Asokan R. (2017). Heteroplasmy due to coexistence of mtCOI haplotypes from different lineages of the *Thrips tabaci* cryptic species group. Bull. Entomol. Res..

[71] Nault B.A., Kain W.C., Wang P. (2014). Seasonal changes in *Thrips tabaci* population structure in two cultivated hosts. PLoS ONE.

[72] Kadirvel P., Srinivasan R., Hsu Y.-C., Su F.-C., de la Peña R. (2013). Application of cytochrome oxidase I sequences for phylogenetic analysis and identification of thrips species occurring on vegetable crops. J. Econ. Entomol..

[73] Tseng L.Y., Chang N.T., Tseng M.J., Yeh W.B. (2010). Genetic variation of *Thrips tabaci* Lindeman (Thysanoptera: Thripidae) in the Pacific rim. Formos. Entomol..

[74] Srinivasan R., Guo F., Riley D.G., Diffie S., Gitaitis R., Sparks A.N., Jeyaprakash A. (2011). Assessment of variation among *Thrips tabaci* populations from Georgia and Peru based on polymorphisms in mitochondrial cytochrome oxidase I and ribosomal ITS2 sequences. J. Entomol. Sci..

[75] Sojnóczki A., Pájtli R.D., Farkas P., Fail J. (2015). Comparative study of *Thrips tabaci* (Lindeman) cytochrome-c oxidase gene subunit I (COI) sequences data. Bodenkultur.

[76] Li X.W., Fail J., Shelton A.M. (2015). Female multiple matings and male harassment and their effects on fitness of arrhenotokous *Thrips tabaci* (Thysanoptera: Thripidae). Behav. Ecol. Sociobiol..

[77] Sogo K., Miura K., Aizawa M., Watanabe T., Stouthamer R. (2015). Genetic structure in relation to reproduction mode in *Thrips tabaci* (Insecta: Thysanoptera). Appl. Entomol. Zool..

[78] Takeuchi R., Toda S. (2011). Discrimination of two reproductive forms of *Thrips tabaci* by PCR-RFLP, and distribution of arrhenotokous *T. tabaci* in Tottori prefecture. Jpn. J. Appl. Entomol. Zool..

[79] Jacobson A.L., Booth W., Vargo E.L., Kennedy G.G. (2013). *Thrips tabaci* population genetic structure and polyploidy in relation to competency as a vector of *Tomato spotted wilt virus*. PLoS ONE.

[80] Silva R., Hereward J.P., Walter G.H., Wilson L.J., Furlong M.J. (2018). Seasonal abundance of cotton thrips (Thysanoptera: Thripidae) across crop and non-crop vegetation in an Australian cotton producing region. Agric. Ecosyst. Environ..

[81] Macharia I., Backhouse D., Skilton R., Ateka E., Wu S.-B., Njahira M., Maina S., Harvey J. (2015). Diversity of thrips species and vectors of *Tomato spotted wilt virus* in tomato production systems in Kenya. J. Econ. Entomol..

[82] Timm A.E., Stiller M., Frey J.E. (2008). A molecular identification key for economically important thrips species (Thysanoptera: Thripidae) in southern Africa. Afr. Entomol..

[83] Hebert P.D.N., Ratnasingham S., Zakharov E.V., Telfer A.C., Levesque-Beaudin V., Milton M.A., Pedersen S., Jannetta P., DeWaard J.R. (2014). Barcoding Canada Data Release.

[84] Surviliene E., Bernotiene R., Valiuskaite A., Duchovskiene L., Tamosiunas R., Rasiukeviciute N. (2013). The Study of Genetic Differentiation of Thrips tabaci (Insecta: Thysanoptera) and Tetranychus urticae (Acari: Tetranychidae) in Lithuania.

[85] Glover R.H., Collins D.W. (2009). COI DNA Barcodes for the Identification of Thrips.

[86] Eckel C.S., Cho K., Walgenbach J.F., Kennedy G.G., Moyer J.W. (1996). Variation in thrips species composition in field crops and implications for tomato spotted wilt epidemiology in North Carolina. Entomol. Exp. Appl..

[87] Chatzivassiliou E.K., Peters D., Katis N.I. (2007). The role of weeds in the spread of *Tomato spotted wilt virus* by *Thrips tabaci* (Thysanoptera: Thripidae) in tobacco Crops. J. Phytopathol..

[88] Marullo R., Grazia A. (2012). Notes on sex ratio and reproductive modes in field populations of two pest thrips species (Thysanoptera, Thripidae) in Italy. Acta Phytopathol. Entomol. Hung..

[89] Sakimura K. (1932). Life history of *Thrips tabaci* L. on *Emilia sagittata* and its host plant range in Hawaii. J. Econ. Entomol..

[90] van Rijn P.C.J., Mollema C., Steenhuis-Broers G.M. (1995). Comparative life history studies of *Frankliniella occidentalis* and *Thrips tabaci* (Thysanoptera: Thripidae) on cucumber. Bull. Entomol. Res..

[91] Tedeschi R., Ciuffo M., Mason G., Roggero P., Tavella L. (2001). Transmissibility of four tospoviruses by a thelytokous population of *Thrips tabaci* from Liguria, northwestern Italy. Phytoparasitica.

[92] Klein M., Gafni R. (1996). Morphological and molecular variations in thrips population collected on onion plants in Israel. Folia Entomol. Hung..

[93] Murai T., Hoshi M., Yamashita O. (1990). Parthenogenetic reproduction in *Thrips tabaci* and *Frankliniella intonsa* (Insecta: Thysanoptera). Advances in Invertebrate Reproduction.

[94] Aizawa M., Watanabe T., Kumano A., Tamagaki K., Sonoda S. (2018). Biotic performances of thelytokous and arrhenotokous strains of *Thrips tabaci* (Thysanoptera: Thripidae) showing resistance to cypermethrin. Appl. Entomol. Zool..

[95] Almási A., Tóbiás I., Bujdos L., Jenser G. (2016). Molecular characterisation of *Thrips tabaci* Lindeman, 1889 (Thysanoptera: Thripidae) populations in Hungary based on the ITS2 sequences. Acta Zool. Acad. Sci. Hung..

[96] Monteiro R.C., Mound L.A., Zucchi R.A. (1999). Thrips (Thysanoptera) as pests of plant production in Brazil. Rev. Bras. Entomol..

[97] Etienne J., Ryckewaert P., Michel B. (2015). Thrips (Insecta: Thysanoptera) of Guadeloupe and Martinique: Updated check-list with new information on their ecology and natural enemies. Fla. Entomol..

[98] Loredo Varela R.C. (2019). Hungarian University of Agriculture and Life Sciences.

[99] Bhatti J.S. (1980). Species of the genus *Thrips* from India (Thysanoptera). Syst. Entomol..

[100] Mound L.A., Palmer J.M. (1973). Notes on Thysanoptera from Israel. Entomol.’s Mon. Mag..

[101] Priesner H. (1919). Zur thysanopteren-fauna Albaniens. Sitz. Kais. Akad. Wiss..

[102] Priesner H. (1920). Beitrag zur kenntnis der thysanopteren Oberösterreichs. Jahrb. Oberösterr. Musealver..

[103] Karadjova O., Hristova D., Adam G. (2001). Epidemiology of tomato spotted wilt tospovirus in Bulgarian tobacco field. Bulg. J. Agric. Sci..

[104] Özden Ö. (2009). The Biodiversity of Invertebrates in Cyprus Ecosystems. Ph.D. Thesis.

[105] Pizzol J., Reynaud P., Bresch C., Rabasse J.M., Biondi A., Desneux N., Parolin P., Poncet C. (2017). Diversity of Thysanoptera species and associated host plants in southern France. J. Mediterr.Ecol..

[106] Boness M., Titschack E., Schliephake G. (1992). Thysanopterenfunde aus Nord- und Westdeutschland. Faun. Ökol. Mitt..

[107] Lindeman K.Ė. (1888). Die schädlichsten insekten des tabak in Bessarabien. Bull. Soc. Imp. Nat. Moscou.

[108] Vierbergen G., Ester A. (2000). Natural enemies and sex ratio of *Thrips tabaci* (Thysanoptera: Thripidae), a major pest of *Allium porrum* in the Netherlands. Med. Fac. Landbouw. Univ. Gent.

[109] Vasiliu-Oromulu L., Marullo R., Mound L.A. (2002). The dynamics of the sex ratio index of thrips populations in mountainous meadows. Proceedings of the 7th International Symposium on Thysanoptera.

[110] Rozhina V.I., Vierbergen G. (2018). Thrips (Thysanoptera) in the meadows of Kaliningrad province. Entomol. Rev..

[111] Torres-Vila L.M., Lacasa A., Bielza P., Meco R. (1994). Dinámica poblacional de *Thrips tabaci* Lind. (Thysanoptera: Thripidae) sobre liliáceas hortícolas en Castilla-La Mancha. Bol. Sanid. Veg. Plagas.

[112] Blunck H. (1958). Thysanopteren aus der Türkei (Thysanoptera). Beitr. Entomol..

[113] Speyer E.R. (1934). Some common species of the genus *Thrips* (Thysanoptera). Ann. Appl. Biol..

[114] Bournier J.P., Mound L.A. (2000). Inventaire commenté des Thysanoptères de Nouvelle-Calédonie. Bull. Soc. Entomol. Fr..

[115] Mound L.A., Masumoto M. (2005). The Genus Thrips (Thysanoptera, Thripidae) in Australia, New Caledonia and New Zealand.

[116] Jenser G., Szénási Á. (2004). Review of the biology and vector capability of *Thrips tabaci* Lindeman (Thysanoptera: Thripidae). Acta Phytopathol. Entomol. Hung..

[117] Kirk W.D.J. (1987). A key to the larvae of some common Australian flower thrips (Insecta, Thysanoptera), with a host-plant survey. Aust. J. Zool..

[118] Kucharczyk H. (2010). Comparative Morphology of the Second Larval Instar of the Thrips Genus Species (Thysanoptera: Thripidae) Occurring in Poland.

[119] Orosz S., Juhász M., Tôkés G., Tóth F. (2008). Occurrence of *Thrips tabaci* larvae on TSWV host weeds in the surroundings of sweet pepper greenhouses. Acta Phytopathol. Entomol. Hung..

[120] Sakimura K. (1939). On the host plants of some Hawaiian thrips. Proc. Hawaii. Entomol. Soc..

[121] de Borbón C. (2007). Clave para la identificación del segundo estadío larval de algunos trips comunes (Thysanoptera: Thripidae). Mendoza, Argentina. Rev. Fac. Cienc. Agrar. Univ. Nac. Cuyo.

[122] Bulger M.A. (1990). Transmission and field spread of *Raspberry bushy dwarf virus*. Plant Dis..

[123] Hardy V.G., Teakle D.S. (1992). Transmission of *Sowbane mosaic virus* by *Thrips tabaci* in the presence and absence of virus-carrying pollen. Ann. Appl. Biol..

[124] Monteiro R.C., Marullo R., Mound L.A. (2002). The Thysanoptera fauna of Brazil. Proceedings of the 7th International Symposium on Thysanoptera.

[125] Miyazaki M., Kudo I. (1986). Descriptions of thrips larvae which are noteworthy on cultivated plants (Thysanoptera: Thripidae). I. Species occurring on solanaceous and cucurbitaceous crops. Akitu.

[126] Blanton F.S. (1939). Notes on some thrips collected in the vicinity of Babylon, Long Island, NY. J. N. Y. Entomol. Soc..

[127] Speyer E.R., Parr W.J. (1941). The external structure of some thysanopterous larvae. Trans. R. Entomol. Soc. Lond..

[128] Sakimura K. (1937). A survey of host ranges of thrips in and around Hawaiian pineapple fields. Proc. Hawaii. Entomol. Soc..

[129] Franssen C.J.H., Mantel W.P. (1965). *Thrips tabaci* op hyacintebollen. Neth. J. Plant Pathol..

[130] Morison G.D. (1948). Thysanoptera of the London area, part II. Lond. Nat..

[131] Thungrabeab M., Blaeser P., Sengonca C. (2006). Effect of temperature and host plant on the efficacy of different entomopathogenic fungi from Thailand against *Frankliniella occidentalis* (Pergande) and *Thrips tabaci* Lindeman (Thysanoptera: Thripidae) in the laboratory. J. Plant Dis. Prot..

[132] Belaam-Kort I. (2020). Etude des Thrips (Thysanoptera) en Vergers d’agrumes en Tunisie en vue de l’établissement d’une stratégie de gestion Raisonnée des Espèces Nuisibles. Ph.D. Thesis.

[133] Carrizo P., Amela García M.T. (2019). Vegetación espontánea en el cinturón hortícola platense hospedante de thripidae (Thysanoptera) vectores de tospovirus: Riesgo relativo como componente epidemiológico. RIA Rev. Investig. Agropecu..

[134] Sakimura K. (1946). Two species of thrips non-vectors of the spotted wilt virus. J. Econ. Entomol..

[135] Orosz S. (2012). Hajtatott Paprika Állományokban és Azok Környezetében élő Thysanoptera Populációk Vizsgálata. Ph.D. Thesis.

[136] Przybylska A., Fiedler Ż., Kucharczyk H., Obrępalska-Stęplowska A. (2015). Detection of the quarantine species *Thrips palmi* by loop-mediated isothermal amplification. PLoS ONE.

[137] Goldarazena A., Loomans A.J.M., Jordama R., Vierbergen G., Tunç I. (1999). Parasitic and parasitoid enemies of thrips (Insecta, Thysanoptera) in northern Spain, an introduction. Proceedings of the Sixth International Symposium on Thysanoptera.

[138] Nischwitz C., Srinivasan R., Sundaraj S., Mullis S.W., McInnes B., Gitaitis R.D. (2012). Geographical distribution and survival of *Iris yellow spot virus* in spiny sowthistle, *Sonchus asper*, in Georgia. Plant Dis..

[139] Gerdes C. (1979). Thysanoptera associated with horseradish in Illinois. Entomol. News.

[140] Burgess L., Weegar H.H. (1988). Thrips (Thysanoptera) in canola crops in Saskatchewan. Can. Entomol..

[141] Franssen C.J.H., Mantel W.P. (1962). Lijst van in Nederland aangetroffen Thysanoptera, met beknopte aantekeningen over hun levenswijze en hun betekenis voor onze cultuurgewassen. Tijdschr. Entomol..

[142] Holdaway F.G., Jones W.W., Storey W.B., Parris G.K., Holdaway F.G. (1941). Insect pests of papaya and their control. Papaya Production in the Hawaiian Islands.

[143] Ábrahám R. (2008). Thrips species associated with soybean in Hungary. Acta Phytopathol. Entomol. Hung..

[144] Hurej M., Kucharczyk H., Twardowski J.P., Kozak M. (2014). Thrips (Thysanoptera) associated with narrow-leafed lupin (*Lupinus angustifolius* L., 1753) intercropped with spring triticale (x *Triticosecale* Wittm. ex A. Camus, 1927). Rom. Agric. Res..

[145] Amin P.W., Palmer J.M. (1985). Identification of groundnut Thysanoptera. Trop. Pest Manag..

[146] Kritzman A., Beckelman H., Alexandrov S., Cohen J., Lampel M., Zeidan M., Raccah B., Gera A. (2000). lisianthus leaf necrosis: A new disease of lisianthus caused by *Iris yellow spot virus*. Plant Dis..

[147] Hurej M., Kucharczyk H., Twardowski J.P., Kotecki A. (2017). Thrips (Thysanoptera) associated with two genetically modified types of linseed (*Linum usitatissimum* L.). J. Plant Dis. Prot..

[148] Takahashi A., Yamauchi T. (1986). Biology of thrips which attack the inside of fig fruits (*Ficus carica* L.). Bull. Shizuoka Pref. Citrus Exp. Sta..

[149] North R.C., Shelton A.M. (1986). Overwintering of the onion thrips, *Thrips tabaci* (Thysanoptera: Thripidae), in New York. Environ. Entomol..

[150] Zawirska I., Walkowski W. (2000). Fauna and importance of thrips (Thysanoptera) for rye and winter wheat in Poland. Part I. J. Plant Prot. Res..

[151] Nielsen H., Sigsgaard L., Kobro S., Jensen N.L., Jacobsen S.K. (2021). Species composition of thrips (Thysanoptera: Thripidae) in strawberry high tunnels in Denmark. Insects.

[152] de Borbón C.M., Becerra V., Bonomo V., Mazzitelli E., Calvo M. (2008). Trips (Insecta: Thysanoptera) en montes de cerezo en Mendoza, Argentina. Rev. Fac. Cienc. Agrar. Univ. Nac. Cuyo.

[153] Tsuchiya M. (2002). Infestation and oviposition of *Thrips tabaci* (Lindeman) on Satsuma mandarin (*Citrus unshiu* Marc.). Jpn. J. Appl. Entomol. Zool..

[154] Navarro-Campos C., Aguilar A., Garcia-Marí F. (2012). Aggregation pattern, sampling plan, and intervention threshold for *Pezothrips kellyanus* in citrus groves. Entomol. Exp. Appl..

[155] Madadi H., Kharazi-Pakdel A., Ashouri A., Neyshabouri J.M. (2006). Life history parameters of *Thrips tabaci* (Thys.: Thripidae) on cucumber, sweet pepper and eggplant under laboratory conditions. J. Entomol. Soc. Iran.

