# Correction: Loredo Varela, R.C.; Fail, J. Host Plant Association and Distribution of the Onion Thrips, *Thrips tabaci* Cryptic Species Complex. *Insects* 2022, *13*, 298

**DOI:** 10.3390/insects13050442

**Published:** 2022-05-07

**Authors:** Roberto Carlos Loredo Varela, József Fail

**Affiliations:** Department of Entomology, Institute of Plant Protection, Hungarian University of Agriculture and Life Sciences, 44 Menesi ut, 1118 Budapest, Hungary; rloredovarela@hotmail.com

## Error in Table

In the original publication [1], there was a mistake in Table 3. The confirmed host plants of onion thrips are as published. For some entries, the host plants were not arranged following alphabetical order within the respective botanical family. Consequently, due to the broken sequence of host plant taxa it was hard for the readers to follow and understand this table. The reference numbers have now been also updated accordingly due to the rearrangement of Table 3. The corrected Table 3 appears below. The authors apologize for any inconvenience caused and state that the scientific conclusions are unaffected. The original publication has also been updated.

**Table 3:** Confirmed host plants of onion thrips.

Plant Species	References
**Aizoaceae**	-
*Mesembryanthemum forskahlii* Hochst. ex Boiss.	[23]
**Alstroemeriaceae**	-
*Alstroemeria* sp. ^†,‡,§,¶^	[8]
**Amaranthaceae**	-
*Amaranthus albus* L. ^†^	[89]
*A. caudatus* L. ^†^	[23]
*A. retroflexus* L. ^†,‡^	[7,22,89]
*Atriplex hortensis* L.	[22]
*A. sagittata* Borkh.	[22]
*A. tatarica* L.	[119]
*Beta vulgaris* L. ^†^	[22,120]
*Chenopodium album* L. ^†,‡^	[22,23,121]
*C. murale* L. ^†^	[23]
*C. quinoa* Willd. ^†,‡,§^	[122]
*C. urbicum* L. ^†^	[28]
*Dysphania ambrosioides* (L.) Mosyakin and Clemants ^†^	[23]
*Spinacia oleracea* L. ^†^	[22,120]
**Amaryllidaceae**	-
*Allium ampeloprasum* L. ^†,‡^	[10,21,108]
*A. ascalonicum* L.	[123]
*A. cepa* L. ^†,‡^	[21,38,43]
*A. fistulosum* L. ^‡^	[94,124,125]
*A. oleraceum* L.	[22]
*A. sativum* L. ^†,‡^	[22,111,124]
*A. schoenoprasum* L. ^‡^	[28]
*Allium* sp.	[9]
*Narcissus* sp.	[126]
**Apiaceae**	-
*Ammi majus* L. ^†^	[21,23,80]
*Anethum graveolens* L.	[112]
*Anthriscus sylvestris* (L.) Hoffm.	[118]
*Apium graveolens* L. ^†^	[120]
*Conium maculatum* L. ^†^	[52]
*Daucus carota* L. ^†^	[89]
*D. sativus* Roehl.	[22]
*Daucus* sp.	[112]
*Petroselinum crispum* (Mill.) Fuss	[22,127]
**Apocynaceae**	-
*Asclepias syriaca* L.	[17,52]
**Araceae**	-
*Arum* sp. ^†,§^	[113,127]
**Araliaceae**	-
*Polyscias guilfoylei* L.H.Bailey	[89]
*Schefflera* sp.	[8]
**Asparagaceae**	-
*Asparagus officinalis* L.	[124,128]
*Asparagus* sp.	[24]
*Hyacinthus* sp.	[129]
*Yucca* sp.	[89]
**Asteraceae**	-
*Acanthospermum australe* (Loefl.) Kuntze	[89]
*Achillea clypeolata* Sm.	[22]
*A. millefolium* L. ^†^	[22,24,28]
*A. nobilis* L.	[22]
*A. ptarmica* L.	[24]
*A. salicifolia* Besser	[118]
*Achillea* spp.	[130]
*Ageratum conyzoides* (L.) L. ^†^	[23,89,128]
*Ambrosia artemisiifolia* L. ^†^	[7,17,119]
*A. maritima* L.	[21]
*Anthemis arvensis* L. ^†^	[7,22,111]
*A. melampodina* Delile	[21,23]
*A. retusa* Delile	[23]
*Arctium minus* (Hill) Bernh. ^†,‡^	[52]
*Arctotheca calendula* (L.) Levyns ^†^	[80]
*Artemisia princeps* Pamp.	[125]
*A. vulgaris* L. ^†^	[24]
*Aster* spp. ^†^	[24]
*Bidens pilosa* L. ^†^	[80,89,128]
*Brachyscome iberidifolia* Benth. ^†^	[131]
*Calendula arvensis* (Vaill.) L. ^†^	[132]
*C. officinalis* L. ^†^	[59]
*Callistephus chinensis* (L.) Nees ^†^	[128]
*Carduus acanthoides* L. ^†^	[22,133]
*C. pycnocephalus* L. ^†^	[22]
*Carlina vulgaris* L.	[118]
*Centaurea calcitrapa* L. ^†^	[23]
*C. cyanus* L. ^†^	[22]
*Chrysanthemum* spp. ^†,‡,§^	[24,59,89]
*Cichorium endivia* L. ^†^	[23,120]
*C. intybus* L. ^†,‡^	[23,120,121]
*Cineraria* sp. ^†^	[24,113]
*Cirsium arvense* (L.) Scop. ^†^	[22,52]
*C. syriacum* (L.) Gaertn.	[21,23]
*Cota tinctoria* (L.) J.Gay ^†^	[22]
*Cyanthillium cinereum* (L.) H.Rob.	[89]
*Dahlia pinnata* Cav.	[22]
*Dahlia* sp. ^†,§^	[8]
*Emilia coccinea* (Sims) G.Don ^†^	[89,128]
*E. sonchifolia* (L.) DC. Ex DC. ^†,§,¶^	[37,134]
*Emilia* spp. ^†^	[128]
*Erigeron annuus* (L.) Pers.	[135]
*Erigeron bonariensis* L. ^†^	[23]
*E. canadensis* L. ^†,‡^	[22,89,128]
*Eupatorium cannabinum* L. ^†^	[22]
*Galinsoga parviflora* Cav. ^†^	[7,17,119]
*Helianthus annuus* L. ^†,‡^	[22]
*Helminthotheca echioides* (L.) Holub ^†^	[133]
*Hypochaeris radicata* L.	[24]
*Inula candida* (L.) Cass.	[22]
*I. helenium* L. ^†^	[22]
*I. salicifolia* Gueldenst.	[136]
*Jacobaea maritima* (L.) Pelser and Meijden ^†^	[22]
*Lactuca sativa* L. ^†,§^	[22,121,128]
*L. serriola* L. ^†,‡^	[64,87]
*Laphangium luteoalbum* (L.) Tzv.	[23]
*Launaea nudicaulis* (L.) Hook.f.	[23]
*Leucanthemum vulgare* Lam. ^†^	[8,22]
*Matricaria chamomilla* L. ^†^	[22,121,133]
*Matricaria* spp.	[24]
*Pluchea indica* (L.) Less.	[89]
*Pulicaria arabica* (L.) Cass.	[23]
*Santolina chamaecyparissus* L.	[137]
*Senecio cruentus* (L’Hér.) DC. ^†,§^	[12]
*S. glaucus* subsp. *coronopifolius* C. Alex.	[21]
*S. pogonias* Cabrera	[121]
*S. umbrosus* Waldst. and Kit.	[118]
*S. viscosus* L.	[24]
*S. vulgaris* L. ^†,‡^	[119,135]
*Senecio* spp. ^†^	[24,130]
*Silybum marianum* (L.) Gaertn. ^†^	[23]
*Solidago canadensis* L. ^†^	[52]
*S. gigantea* Aiton	[118]
*S. virgaurea* L.	[118]
*Solidago* spp. ^†^	[24]
*Sonchus asper* (L.) Hill ^†,‡^	[138]
*S. oleraceus* (L.) L. ^†,‡^	[23,58,80]
*Tanacetum cinerariifolium* (Trevir.) Sch.Bip. ^†^	[21,22]
*T. vulgare* L.	[118]
*Taraxacum officinale* (L.) Weber ex F.H.Wigg. ^†,‡^	[7,28,52]
*Tithonia rotundifolia* (Mill.) S.F.Blake ^†^	[89]
*Tragopogon dubius* Scop. ^‡^	[64]
*T. porrifolius* L. ^†^	[22]
*Tripleurospermum indorum* (L.) Sch.Bip. ^†^	[22]
*T. maritmum* (L.) Koch	[119]
*Verbesina encelioides* (Cav.) Benth. and Hook.f. ex A.Gray ^†^	[55]
*Xanthium strumarium* L. ^†^	[80]
*Zinnia elegans* L. ^†^	[22]
**Balsaminaceae**	-
*Impatiens* sp. ^†,‡,§,¶^	[45]
**Boraginaceae**	-
*Anchusa azurea* Mill. ^†^	[111]
*Bothriospermum tenellum* (Hornem.) Fisch. and Mey.	[89]
*Echium plantagineum* L.	[58,80,133]
*Heliotropium europaeum* L. ^†^	[22]
**Brassicaceae**	-
*Alyssum* spp.	[26]
*Armoracia rusticana* P.Gaertn., B.Mey. and Scherb.	[139]
*Barbarea vulgaris* R.Br. ^†^	[52]
*Berteroa incana* (L.) DC.	[22]
*Biscutella auriculata* L.	[111]
*Brassica cretica* Lam.	[22]
*B. juncea* (L.) Czern.	[140]
*B. napus* L. ^†^	[80]
*B. nigra* (L.) K.Koch	[89]
*B. oleracea* L. ^†^	[21,38,60]
*B. rapa* L. ^†^	[22,133]
*Brassica* sp.	[125]
*Capsella bursa-pastoris* (L.) Medik. ^†^	[52,80,121]
*Cochlearia* sp.	[24]
*Descurainia sophia* (L.) Webb ex Prantl ^‡^	[64]
*Diplotaxis* sp.	[111]
*Erophila verna* (L.) DC.	[22]
*Eruca vesicaria* (L.) Cav.	[21,23,121]
*Erysimum* sp.	[24]
*Lepidium bonariense* L.	[80]
*L. didymum* L. ^†^	[89]
*L. draba* L. ^†^	[22,135]
*L. latifolium* L.	[22]
*L. virginicum* L. ^†^	[52]
*Lobularia* spp.	[26]
*Raphanus raphanistrum* subsp. *sativus* (L.) Domin	[23,89,133]
*R. raphanistrum* L. ^†^	[52]
*Raphanus* sp.	[141]
*Rapistrum rugosum* (L.) All.	[58,80,133]
*Sinapis alba* L.	[112]
*S. arvensis* L. ^†,‡^	[22,52]
*Sisymbrium irio* L.	[21,23,121]
*S. officinale* (L.) Scop.	[22]
*Sisymbrium* sp.	[111]
*Thlaspi arvense* L.	[52]
**Bromeliaceae**	-
*Ananas comosus* (L.) Merr. ^†^	[89,128]
**Cactaceae**	-
*Opuntia* sp. ^†^	[24]
**Calceolariaceae**	-
*Calceolaria* sp. ^†^	[127]
**Campanulaceae**	-
*Campanula* sp. ^†^	[24]
**Caricaceae**	-
*Carica papaya* L. ^†^	[120,142]
**Caryophyllaceae**	-
*Dianthus barbatus* L.	[8]
*D. campestris* M.Bieb.	[22]
*D. caryophyllus* L.	[21,59]
*D. corymbosus* Sm.	[22]
*Dianthus* spp. ^†^	[8,24,89]
*Spergularia rubra* (L.) J.Pr. and C.Pr.	[24]
*Stellaria media* (L.) Vill. ^†^	[7,51,119]
**Commelinaceae**	-
*Commelina* sp.	[120]
*Murdannia nudiflora* (L.) Brenan	[120]
*Tradescantia* sp. ^†^	[24]
**Convolvulaceae**	-
*Calystegia sepium* (L.) R. Br. ^†^	[52]
*Convolvulus arvensis* L. ^†,‡^	[7,21,121]
*Ipomoea batatas* (L.) Lam. ^†^	[22,125]
*I. indica* (Burm.) Merr. ^†^	[128]
*Operculina aequisepala* (Domin)	[80]
**Crassulaceae**	-
*Sedum acre* L.	[118]
*Sedum* sp. ^†^	[24]
**Cucurbitaceae**	-
*Citrullus lanatus* (Thunb.) Matsum. and Nakai ^†^	[21,22]
*Cucumis melo* L. ^†^	[22]
*C. sativus* L. ^†,§^	[8,90,125]
*Cucurbita maxima* Duchesne ^†^	[22]
*C. moschata* Duchesne ^†^	[21,22]
*C. pepo* L. ^†^	[21,22]
**Ebenaceae**	-
*Diospyros kaki* L.f.	[14]
**Ericaceae**	-
*Ledum palustre* L.	[118]
**Euphorbiaceae**	-
*Chrozophora tinctoria* (L.) A.Juss.	[21]
*Euphorbia hirta* L.	[89]
*Ricinus communis* L. ^†,‡^	[23]
**Fabaceae**	-
*Anthyllis vulneraria* L.	[118]
*Anthyllis* sp.	[24]
*Arachis hypogaea* L. ^†^	[22]
*Astragalus cicer* L.	[22]
*Bauhinia* sp.	[23]
*Cajanus cajan* (L.) Millsp. ^†^	[89]
*Cicer arietinum* L. ^†^	[117]
*Crotalaria juncea* L. ^†^	[89]
*C. saltiana* Andrews	[89]
*C. spectabilis* Roth ^†^	[89]
*Cytisus nigricans* L.	[135]
*Delonix regia* (Boj.ex Hook.) Raf.	[21]
*Galega officinalis* L.	[133]
*Glycine max* (L.) Merr. ^†,§^	[22,143]
*Hippocrepis emerus* (L.) Lassen	[22]
*Lathyrus* sp.	[24]
*Lens culinaris* Medik ^†^	[21]
*Lotus corniculatus* L.	[22]
*L. tenuis* Waldst. and Kit.	[133]
*Lotus* sp.	[24]
*Lupinus angustifolius* L. ^†^	[144]
*Lupinus* sp. ^†^	[89]
*Medicago lupulina* L. ^†^	[22,52]
*M. polymorpha* var. *vulgaris* (Benth.) Shinners	[80]
*M. sativa* L.	[21,80,89]
*Melilotus albus* Medik.	[118]
*M. indicus* (L.) All. ^†^	[21,23]
*M. messanensis* (L.) All.	[23]
*M. officinalis* (L.) Pall. ^†^	[22,119]
*Mimosa pudica* L.	[89]
*Ornithopus compressus* L.	[22]
*Phaseolus vulgaris* L. ^†,¶^	[21,22,121]
*Pisum sativum* L. ^†,§^	[89,117,128]
*P. sativum* subsp. *elatius* (M.Bieb.) Asch. and Graebn.	[22]
*Senna occidentalis* (L.) Link ^†^	[89]
*Trifolium alexandrinum* L.	[21,112]
*T. pratense* L.	[22,112]
*T. repens* L. ^†^	[7,22,133]
*Trifolium resupinatum* L.	[23]
*Trigonella foenum-graecum* L.	[21]
*T. laciniata* L.	[23]
*Vicia faba* L. ^†,§,¶^	[21,77,80]
*V. sativa* L. ^‡^	[80,121]
*Vigna mungo* (L.) Hepper ^†^	[145]
*V. unguiculata* subsp. *unguiculata* (L.) Walp. ^†^	[89]
**Gentianaceae**	-
*Eustoma russellianum* (Hook.) G.Don ^†,‡^	[146]
**Geraniaceae**	-
*Erodium cicutarium* (L.) L’Hér. ^‡^	[22]
*Geranium pratense* L.	[118]
*G. pusillum* L.	[22]
*G. pyrenaicum* Burm.f.	[22]
**Gesneriaceae**	-
*Saintpaulia brevipilosa* B.L.Burtt	[131]
**Hypericaceae**	-
*Hypericum perforatum* L.	[22]
**Iridaceae**	-
*Freesia* sp.	[8]
*Gladiolus* spp. ^†^	[8]
**Juncaginaceae**	-
*Triglochin maritima* L.	[24]
**Lamiaceae**	-
*Glechoma hederacea* L.	[22,28]
*Lamium album* L.	[28]
*L. amplexicaule* L. ^†^	[7,23,121]
*L. purpureum* L. ^†^	[22,52]
*Lamium* spp.	[28]
*Nepeta cataria* L. ^†^	[52]
*Nepeta* spp.	[24]
*Origanum vulgare* L.	[121]
*Rosmarinus* spp.	[26]
*Salvia* sp. ^†^	[24]
*Teucrium scorodonia* L.	[24]
**Linaceae**	-
*Linum usitatissimum* L.	[147]
**Lythraceae**	-
*Cuphea hyssopifolia* Kunth	[89]
**Malvaceae**	-
*Abutilon theophrasti* Medik.	[22]
*Alcea rosea* L.	[21,22]
*Althaea officinalis* L. ^†^	[22]
*Althaea* sp. ^†,§^	[24]
*Gossypium arboreum* L.	[22]
*Gossypium barbadense* L. ^†^	[22]
*G. hirsutum* L. ^†^	[22,80]
*Gossypium* spp.	[21]
*Malva neglecta* Wallr. ^†^	[22,52,64]
*M. parviflora* L. ^†^	[121]
*M. sylvestris* L. ^†^	[22]
*Malva* sp. ^†^	[89]
*Sphaeralcea miniata* (Cav.) Spach	[121]
*Tilia cordata* Mill.	[22]
*T. tomentosa* Moench	[22]
*Waltheria indica* L.	[89]
**Moraceae**	-
*Ficus carica* L.	[148]
*Morus alba* L.	[21,22]
*M. nigra* L.	[22]
**Myrtaceae**	-
*Eucalyptus* spp.	[26]
**Nyctaginaceae**	-
*Boerhavia glabrata* Blume	[80]
**Onagraceae**	-
*Oenothera biennis* L. ^†^	[52]
**Oxalidaceae**	-
*Oxalis corniculata* L. ^†^	[23]
*O. debilis* Kunth	[89]
*O. stricta* L. ^†^	[52]
*Oxalis* sp. ^†^	[24]
**Papaveraceae**	-
*Chelidonium majus* L.	[28]
*Fumaria capreolata* L.	[80]
*Papaver rhoeas* L. ^†^	[111]
**Phytolaccaceae**	-
*Phytolacca acinosa* Roxb.	[89]
**Plantaginaceae**	-
*Digitalis* sp. ^†^	[24]
*Linaria vulgaris* Mill.	[22,112]
*Plantago lanceolata* L. ^†,‡^	[52,121]
*P. major* L. ^†^	[28]
*P. maritima* L.	[24]
*Veronica austriaca* L.	[22]
*V. persica* Poir. ^†^	[121]
**Plumbaginaceae**	-
*Armeria* sp.	[24]
**Poaceae**	-
*Avena fatua* L. ^†^	[80]
*A. sativa* L.	[149]
*Avena* sp.	[52]
*Bromus inermis* Leyss. ^‡^	[28]
*Cenchrus americanus* (L.) Morr.	[22]
*Dactylis glomerata* L.	[111]
*Digitaria sanguinalis* (L.) Scop. ^†^	[22,89]
*D. violascens* Link	[89]
*Eleusine indica* (L.) Gaertn. ^†,‡^	[89]
*Elymus repens* (L.) Gould	[28]
*Hordeum maritimum* With.	[21,23]
*H. vulgare* L.	[21,60,149]
*Lolium* sp.	[52]
*Phleum pratense* L.	[149]
*Poa annua* L. ^†^	[28]
*Polypogon monspeliensis* (L.) Desf	[23]
*P. viridis* (Gouan) Breistr.	[23]
*Secale cereale* L.	[150]
*Setaria verticillata* (L.) P.Beauv.	[22]
*S. viridis* (L.) P.Beauv. ^†,‡^	[22]
*Sorghum halepense* (L.) Pers.	[22]
*Triticum aestivum* L. ^‡^	[58,149,150]
**Polygonaceae**	-
*Persicaria maculosa* Gray ^†^	[22]
*P. orientalis* (L.) Spach	[80]
*Polygonum aviculare* L. ^†^	[80]
*Rumex crispus* L. ^†,‡^	[58,80]
*R. dentatus* L.	[23]
*R. obtusifolius* L.	[119]
**Portulacaceae**	-
*Portulaca oleracea* L. ^†,‡,§^	[89]
**Primulaceae**	-
*Cyclamen coum* Mill.	[22]
*Cyclamen* spp. ^†^	[8,127]
**Ranunculaceae**	-
*Helleborus odorus* Waldst. and Kit.	[22]
*Ranunculus arvensis* L. ^†^	[22]
*R. polyanthemos* L.	[22]
**Resedaceae**	-
*Reseda* spp.	[26]
**Rosaceae**	-
*Fragaria × ananassa* (Duchesne ex Weston) Duchesne ex Rozier	[151]
*Malus domestica* Borkh.	[22]
*Potentilla alba* L.	[22]
*Prunus avium* (L.) L.	[152]
*P. domestica* L.	[22]
*P. persica* (L.) Batsch	[24]
*Prunus* spp.	[26]
*Pyrus communis* L.	[22]
*Rosa canina* L.	[22]
*R. damascena* Mill.	[22]
*Rosa* sp. ^†,‡,§^	[9,127]
**Rubiaceae**	-
*Galium aparine* L. ^†^	[22]
*G. verum* L. ^†^	[118,119]
*Galium* sp. ^†^	[24]
*Richardsonia scabra* L. ^†^	[89,128]
**Rutaceae**	-
*Citrus unshiu* (Yu.Tanaka ex Swingle) Marcow	[153]
*Citrus* spp.	[154]
**Salicaceae**	-
*Salix mucronata* Thunb.	[21]
*Salix* sp.	[23]
**Scrophulariaceae**	-
*Verbascum lychnitis* L.	[118]
*V. thapsus* L. ^†^	[52]
**Solanaceae**	-
*Alkekengi officinarum* Moench ^†^	[22]
*Atropa bella-donna* L. ^†^	[22]
*Capsicum annuum* L. ^†,‡,§^	[120,155]
*Datura stramonium* L. ^†,‡,§,¶^	[28,45,120]
*Hyoscyamus niger* L. ^†^	[22]
*Lycium barbarum* L.	[22]
*Nicandra physalodes* (L.) Gaertn. ^†^	[89]
*Nicotiana glauca* Graham ^†^	[128]
*N. rustica* L. ^†,‡,§^	[22]
*N. tabacum* L. ^†,§,¶^	[10,28,107]
*Nicotiana* sp. ^†^	[112]
*Petunia hybrida* Vilm ^†,‡,§^	[22]
*Solanum americanum* Mill. ^†^	[89,128]
*S. dulcamara* L. ^†^	[22]
*S. lycopersicum* L. ^†,‡,§^	[21–23]
*S. melongena* L. ^†,§^	[22,120,155]
*S. nigrum* L. ^†,‡^	[22,28,87]
*S. tuberosum* L. ^†,‡,§^	[21,28,120]
**Tropaeolaceae**	-
*Tropaeolum majus* L. ^†,§^	[22]
**Urticaceae**	-
*Urtica dioica* L. ^†^	[52]
**Verbenaceae**	-
*Lantana camara* L.	[89]
*Pitraea cuneato-ovata* (Cav.) Caro	[121]
*Stachytarpheta cayennensis* (Rich.) Vahl	[89]
*Verbena bonariensis* L.	[89]
*V. officinalis* L. ^†^	[80]
*V. tenuisecta* Briq.	[58]
**Viburnaceae**	-
*Sambucus ebulus* L.	[22]
**Vitaceae**	-
*Vitis vinifera* L.	[22]
**Zygophyllaceae**	-
*Tribulus terrestris* L. ^†,‡^	[22,80]

Note: Plant species known to host: ^†^ Tomato Spotted Wilt Virus (TSWV) [62,63]; ^‡^ Iris Yellow Spot Virus (IYSV) [11,52,63–65]; ^§^ Tomato Yellow Ring Virus (TYRV) [12,63,66]; and ^¶^ Alstroemeria Yellow Spot Virus (AYSV) [13]. - = There are no data.
